# Neural connectivity molecules best identify the heterogeneous clock and dopaminergic cell types in the *Drosophila* adult brain

**DOI:** 10.1126/sciadv.ade8500

**Published:** 2023-02-22

**Authors:** Dingbang Ma, Nicholas Herndon, Jasmine Quynh Le, Katharine C. Abruzzi, Kai Zinn, Michael Rosbash

**Affiliations:** ^1^ Howard Hughes Medical Institute and Department of Biology, Brandeis University, Waltham, MA 02454, USA.; ^2^ Division of Biology and Biological Engineering, California Institute of Technology, Pasadena, CA 91125, USA.

## Abstract

Our recent single-cell sequencing of most adult *Drosophila* circadian neurons indicated notable and unexpected heterogeneity. To address whether other populations are similar, we sequenced a large subset of adult brain dopaminergic neurons. Their gene expression heterogeneity is similar to that of clock neurons, i.e., both populations have two to three cells per neuron group. There was also unexpected cell-specific expression of neuron communication molecule messenger RNAs: G protein–coupled receptor or cell surface molecule (CSM) transcripts alone can define adult brain dopaminergic and circadian neuron cell type. Moreover, the adult expression of the CSM *DIP-beta* in a small group of clock neurons is important for sleep. We suggest that the common features of circadian and dopaminergic neurons are general, essential for neuronal identity and connectivity of the adult brain, and that these features underlie the complex behavioral repertoire of *Drosophila.*

## Teaser

Connectivity molecule transcript expression can define neuron type in the adult fly brain.

## INTRODUCTION

The fruit fly *Drosophila melanogaster* has a sophisticated repertoire of behaviors. Together with its remarkable genetic toolkit and tiny brain, it is an ideal model organism for studying the neuroscience of behavior. Somewhat at odds with this simplicity, however, is the extraordinary complexity of wiring and gene expression patterns of adult fly brain neurons. Analysis of the relationships between wiring and gene expression is fundamental to understanding how these neurons form circuits and function to regulate behavior.

The fly brain wiring patterns have been illuminated by recent advances in genetics as well as light and electron microscopy (EM) (*1*), whereas advances in single-cell RNA sequencing technologies have provided unprecedented insight into neuronal gene expression regulation (*2*). These technologies have also been applied to many large groups of fly neurons, including optical lobes and whole-brain profiling (*3*–*8*). These studies have led to the characterization of cellular plasticity during development and the de novo identification of cell type (*9*, *10*), and they include a recent global collaboration effort, the Fly Cell Atlas project, to produce cellular gene expression maps of the entire fly (*8*).

Single-cell RNA sequencing after neuronal purification has also been applied to many smaller groups of fly brain neurons, including olfactory receptor neurons and olfactory projection neurons (*11*–*14*). This approach has also revolutionized our view of the 150 adult brain circadian neurons (*15*), within which a now well-defined transcription-translation feedback loop generates ~24-hour periodicity (*16*).

These circadian neurons have historically been grouped based on anatomy. There are ventral lateral neurons, dorsal lateral neurons, and lateral posterior neurons. Four additional clock neuron groups were identified in the dorsal brain, including the DN1ps, DN1as, DN2s, and DN3s (*17*, *18*). Single-cell RNA sequencing of these clock neurons at different times of day revealed notable spatial and temporal gene regulation and substantially expanded the number of clock neuron groups, from about 8 to 10 to at least 17. Many of these high-confidence clock neuron groups or clusters correspond to two or three neurons per hemisphere (*19*). It is, however, uncertain whether this transcriptomic heterogeneity is only true of these ca. 100 clock neurons or is characteristic of other fly central brain neuron groups. This heterogeneity could even be common and contribute to the substantial central brain anatomical heterogeneity indicated by the hemibrain EM connectome (*1*).

To address this issue, we decided to characterize dopaminergic neurons (DANs). Immunohistochemistry of tyrosine hydroxylase (TH), an enzyme required for dopamine synthesis, had identified ~282 neurons per adult fly brain. They were categorized into different anatomical clusters including four anterior groups (PAM, PAL, T1, and Sb) and six major posterior groups (PPL1, PPL2ab, PPL2c, PPM1, PPM2, and PPM3) (*20*). A large body of evidence implicates DANs in the control of many different behaviors including locomotor activity, memory, arousal, aggression, and sleep (*21*–*28*). Consistent with their anatomical and functional diversity, DANs exhibit complex projection patterns: for example, 20 distinct DAN types each project axons to one or at most two mushroom body output neuron compartments (*29*).

In this study, we generated multiple time-point single-cell data from DANs by a modified CEL-seq2 (Cell Expression by Linear amplification and Sequencing 2) method as we had previously done for clock neurons. To minimize batch effects and to apply an additional sequencing strategy, we labeled clock neurons and DANs in the same fly and assayed these two populations together with a droplet-based method (10X Chromium). An unsupervised clustering algorithm identified 43 high-confidence clusters in the pooled dataset, and all of the previously identified clock neuron clusters were matched unambiguously in the current dataset. Using these clock neuron clusters as a benchmark, we show that DANs are comparably heterogeneous. Moreover, cell surface molecules (CSMs) and neuronal connectivity molecules more generally are prominent features of adult brain DAN identity, such as during development of the visual system and projection neurons (*9*–*11*, *13*) and clock neurons (*19*). These data and others suggest that these features may be general properties of neurons within the *Drosophila* central brain.

## RESULTS

### Single-cell sequencing of clock and DANs by plate and droplet methods

We subjected a substantial fraction of DANs to single-cell RNA sequencing. This DAN subset is labeled by TH-GAL4 (*20*) and is comparable in number to the clock neuron subset labeled by Clk856-GAL4 (*30*). Moreover, DANs are arguably the most studied behaviorally relevant neurons in fruit flies. As clock gene expression is very low or absent in DANs (*31*), they might be representative of noncircadian fly brain neurons.

**Fig. 1.**
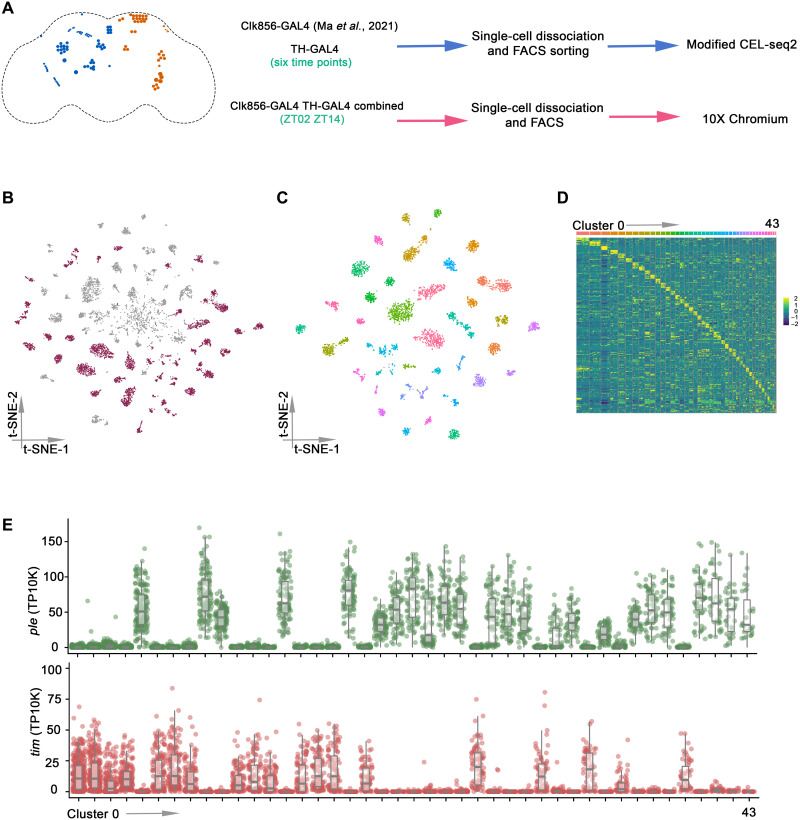
Single-cell RNA sequencing of *Drosophila* clock neurons and DANs by plate and droplet-based methods. (**A**) Schematic workflow of single-cell RNA sequencing data generation. The clock neurons (orange) and DANs (blue) are shown in the schematic depiction on the left panel. Enhanced green fluorescent protein (EGFP)–labeled DANs were sorted into 384-well plates by fluorescence-activated cell sorting (FACS), and single-cell RNA sequencing libraries were prepared by modified CEL-seq2 method. For droplet-based method, TH-GAL4 and Clk856-GAL4 were combined into a stable line to assay DANs and clock neurons simultaneously. Fly brains were dissected and dissociated before droplet encapsulation of individual cells with barcoded beads in 10X Chromium. (**B**) *t*-distributed stochastic neighbor embedding (t-SNE) plot showing the 9025 cells grouped into 70 clusters. High-confidence clusters (see Materials and Methods) are shown in purple. (**C**) t-SNE plot of 4543 *Drosophila* clock and DANs in 43 high-confidence clock and DAN clusters from LD conditions. The clusters are colored by their cell types. (**D**) Heatmap showing the expression levels of the top 5 differentially expressed genes (rows) in cells (columns). Clusters are ordered by size and are represented by different colors on top of the heatmap. (**E**) Dot plot showing *tim* (bottom) and *ple* (top) expression in all clusters. Gene expression levels for each cell were normalized by total expression level and reported by transcripts per 10 thousand transcripts (TP10K). Clusters are ordered by size.

We first characterized DANs with the same modified CEL-seq2 method used for clock neurons (*19*, *32*) and even assayed different times of day by collecting flies at multiple time points (Fig. 1A). Although these data were comparable to previous clock neuron data (*19*), we added an additional approach: TH-GAL4 and Clk856-GAL4 were combined into a stable line (fig. S1A) to profile clock neurons and DANs together; this stable line avoids batch effects. We also collected flies at two LD (light:dark) time points, ZT02 and ZT14, and changed the assay method: The single-cell RNA sequencing of this combined line was done with 10X Chromium chemistry. This provided another point of comparison with the clock neurons, modified CEL-seq2 versus 10X Chromium. The sequencing reads from CEL-seq2 and 10X Chromium were mapped to the *Drosophila* genome (dm6) by zUMIs and Cell Ranger separately (*33*). Only the alignments to annotated exons were counted and used for unique molecular identifier (UMI) quantitation.

To catalog and compare single-cell gene expression in clock neurons and DANs, we combined the single cells from the plate- and droplet-based methods in the current study with the previous CEL-seq2 clock neuron LD data (fig. S1B) (*19*). The single cells were first filtered on the basis of the number of detected genes, transcripts, and gene expression entropy. Possible doublets in 10X Chromium data were identified and excluded from the downstream analysis. A total of 9025 cells remained after this stringent filtering (fig. S1C). Next, single cells were separated by time point and method to identify highly variable genes from each condition; only the common highly variable genes were used for the clustering analysis. Batch effects were removed by this approach, and it produced 70 distinct clusters (Fig. 1B). Despite some variability, cells from both CEL-seq2 and 10X Chromium were present in all clusters (fig. S2A), and similar numbers of genes were identified in most clusters (fig. S2B). This is also because we filtered them to ensure that each cluster has cells from all time points and from both experimental methods (fig. S2, C and D). This was to ensure that the identified clusters are most likely to correspond to specific clock neurons and DANs and that the clusters are minimally contaminated with poor-quality cells (see Materials and Methods). This strategy resulted in 43 high-confidence clusters (Fig. 1C), and each cluster showed expected shared and specific marker gene expression (Fig. 1Dand fig. S2E).

To distinguish between clock and DAN clusters, we characterized the expression of *timeless* (*tim*) and *pale* (*ple*), which are hallmark genes for these two neuron groups, respectively. Consistent with previous results showing that clock gene expression is very low in DANs (*31*), we found that *tim* and *ple* expressions are mutually exclusive, defining 19 clock (*tim^+^*) and 24 DAN (*ple^+^*) clusters (Fig. 1E). Other DAN-positive genes are coexpressed with *ple*, and other clock genes are coexpressed with *tim* (fig. S3). *tim* mRNA is also cycling with its characteristic gene expression peak at ZT14 to ZT18 in all clock clusters (fig. S4). Moreover, genome-wide cycling gene expression analysis identified many cell type–specific cycling transcripts in clock clusters but only tiny number of oscillating transcripts in DAN clusters (fig. S5), consistent with previous results (*31*).

### Identifying clock neuron and DAN clusters

**Fig. 2.**
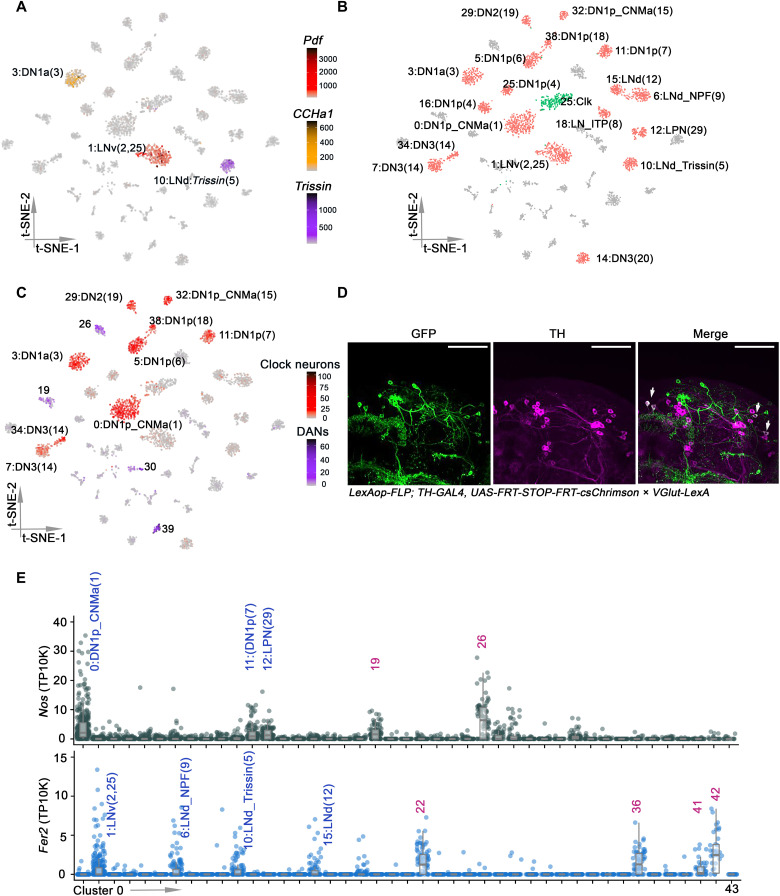
Identifying clock neurons and DANs clusters. (**A**) t-SNE plots showing *Pdf* (red), *Trissin* (purple), and *CCHa1* (yellow) expression in all clusters. Each cell is colored by the expression (color bars, TP10K) with gray indicating low expression and black indicating the highest expression. (**B**) t-SNE visualization of the annotated clock clusters. Each clock neuron cluster retains its original identifying number in the parentheses, as it was previously reported. The clusters in red were all previously identified, and cluster 2 (green) was previously unknown. (**C**) t-SNE plots showing *VGlut* expression in all clusters. Each cell is colored by the expression of *VGlut* (color bars, red and purple represents *VGlut* expression in clock neurons and DANs, respectively, TP10K), with gray indicating low expression and black indicating the highest expression. (**D**) Confocal stack of images showing antibody staining for GFP (left), TH (middle), and the merged image (right) in *VGlut-LexA* > *LexAop-FLP; TH-GAL4* > *UAS-FRT-STOP-FRT-CsChrimson.venus* fly brains. Two PPM1 and two PPL1 neurons are both GFP-positive and TH-positive. Scale bars, 50 μm. (**E**) Dot plot showing *Fer2* (bottom) and *Nos* (top) expression in all clusters. Gene expression levels for each cell were normalized by total expression level and reported by TP10K. Clusters are ordered by size.

Our previous study identified at least 17 high-confidence clock neuron groups with notable spatial and temporal regulation of gene expression (*19*). To correlate these groups with our new dataset, we first characterized the expression of known clock neuron marker genes. For example, the neuropeptide genes *Pigment-dispersing factor* (*Pdf*), *Trissin*, and *CCHamide-1* (*CCHa1*) are expressed in three clusters representing three different clock neuron groups: eight ventral lateral neurons (LNv), two dorsal lateral neurons (LNd), and two DN1a clock neurons, respectively (*19*, *34*, *35*). Each of these neuropeptide transcripts also corresponds to a single clock neuron cluster in the new single-cell RNA sequencing data (Fig. 2A). More generally, this marker gene strategy along with core clock gene expression could match almost all new clock neuron clusters to the previous assignments (Fig. 2B). The only exceptions are the previously unidentified cluster 2, which contains cells from multiple clock neuron groups (fig. S6A), and the previously unknown cluster 25; the latter is an extra DN1p cluster, reflecting two DN1p clusters where there was previously only one.

An interesting marker is the vesicular glutamate transporter *VGlut* mRNA, which identifies glutamatergic neurons. It is expressed in nine dorsal clock neuron clusters (Fig. 2C, red), which corresponds precisely to the prior number of glutamatergic clock neuron groups (*19*, *36*). *VGlut* is also expressed in four dopaminergic clusters (Fig. 2C, purple), consistent with previous findings that glutamate and dopamine are coexpressed in some larval and adult fly brain neurons (*3*, *37*). In contrast to flies, glutamate is the major excitatory neurotransmitter in mammals and is also coexpressed with dopamine in some mammalian neurons (*38*–*40*).

To further verify the expression of *VGlut* in fly DANs, we used a method that allows genetic access to the neurons at the intersection of a LexA and GAL4 line. We expressed UAS>stop>CsChrimson.venus, a FLP-dependent conditional reporter, in TH-GAL4 in addition to LexAop-FLP in a VGlut-LexA knock-in line. Cells that express both the conditional reporter and FLP were then labeled with Cschrimson.venus. CsChrimson.venus expression was restricted to only a few DANs, which include the two PPM1 and two PPL1 neurons (Fig. 2D); they were the strongest and most reproducible double-positive cells and had not been previously identified as glutamatergic.

To further identify DAN clusters, we examined the expression of several known dopaminergic marker genes. Nitric oxide is a cotransmitter in a subset of DANs and acts antagonistically to dopamine (*41*). Bulk RNA sequencing and immunohistochemistry showed that *Nos* (nitric oxide synthase) is expressed in PPL1-γ1pedc and PAM-γ5 neurons (*41*). The data here indicate that *Nos* is expressed only in two DAN clusters (clusters 19 and 26), suggesting that they correspond to PPL1-γ1pedc and PAM-γ5 neurons. As there are only two other glutamate-expressing DAN clusters, 30 and 39, they probably correspond to PPM1 and PPL1 (Fig. 2D). *Nos* mRNA has not been described in clock neurons, but the gene is comparably expressed in three DN1p clock neuron clusters, cluster 0, 11, and 12 (Fig. 2E, top).

*48-related-2* (*Fer2*) encodes a transcription factor (TF), which is expressed in DANs in the PAM cluster and required for their development and survival (*42*). Although immunohistochemistry of TH indicates that there are about 100 PAM DANs, the *TH-GAL4* driver only identifies 13 PAM cells per hemisphere (*20*). As there are four DAN clusters with *Fer2* expression (Fig. 2E, bottom), they presumably correspond to these 13 PAM DANs. This suggests an average of about three PAM DANs per cluster, consistent with subsequent calculations on other clusters (see below). There are apparently four clock clusters that also express *Fer2* (Fig. 2E, bottom).

These marker genes assign eight DAN clusters to known DANs. To assign additional clusters, we compared cluster gene expression with previously published transcriptomes from several different DANs subgroups (*41*). Most of these comparisons were unsuccessful, perhaps because most subgroups were PAMs, and they are very underrepresented by TH-GAL4. However, an otherwise uncharacterized DAN cluster, #28, is highly correlated with PPL1-α3 and PPL1-γ2 α′2 neurons (fig. S6B). This assigns a ninth cluster and hints that this single cluster may not be homogeneous, i.e., that the DANs are even more heterogeneous than indicated by this analysis.

### Neuropeptide expression in clock neurons and DANs

**Fig. 3.**
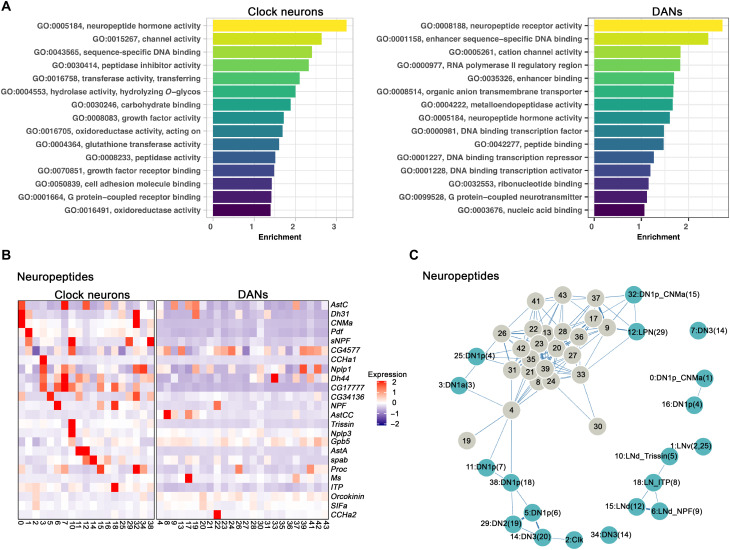
Neuropeptide expression in clock neurons and DANs clusters. (**A**) GO analysis of enriched marker genes found in the clock neuron and DAN clusters. The enriched GO terms were ranked by their relative enrichment. (**B**) Heatmap showing the expression levels of neuropeptides in clock neurons (left) and DANs (right). Red indicates high expression, and purple indicates low expression. (**C**) Gene expression correlation of neuropeptides in clock neurons and DANs. We calculated the Spearman’s correlation coefficients between expression patterns of neuropeptides across different clock neuron and DANs cell types, and the result is visualized in a force-embedded layout. Each cluster is represented by a node with the width of the edge representing the strength of the gene expression.

To further characterize the clock neurons and DANs, we assayed differential gene expression using a negative binomial generalized linear model. Consistent with previous findings, Gene Ontology (GO) analysis indicates that clock neuron transcriptomes are enriched for the term neuropeptide hormone activity (*19*, *31*); it was the top term (Fig. 3A), and many clock neurons express a complex combination of neuropeptides (Fig. 3B). This same term is present in DANs but lower down on its list (Fig. 3A), and only a handful of specific neuropeptides were identified in DANs compared to clock neurons (Fig. 3B).

Nonetheless, four neuropeptides—*Dh44*, *Nplp1*, *Glycoprotein hormone beta 5* (*Gpb5*) and *Proctolin* (*proc*)—have been reported to be expressed in up to 21% of DANs (*4*), but our data indicate that only *Dh44* mRNA is expressed in the TH-Gal4 DANs, in cluster #33 (fig. S7A). *Dh31* mRNA is also enriched in one DAN cluster, #19, and probably corresponds to PPL1-γ1pedc or PAM-γ5 (fig. S7B). *Dh31* is also expressed in the two previously described clock neuron clusters (fig. S7B) (*19*, *43*). The transcript encoding the neuropeptide *Ms* is similarly detected in a single DAN cluster, #17 (fig. S7C).

Several DANs do, however, express more than one neuropeptide mRNA: *AstC* is expressed along with *Ms* in cluster 17 (fig. S7, C and D); *Dh31* and *AstC* are coexpressed in cluster 19 (fig. S7, B and D). Unexpectedly, this likely PPL1-γ1pedc or PAM-γ5 cluster coexpresses glutamate and *Nos* as well as dopamine. Despite these exceptions, neuropeptide transcript expression in the DANs appears limited and may be influenced by incomplete coverage by TH-Gal4 (see Discussion).

To further compare neuropeptide expression between clusters and between clock neurons and DANs, we calculated the Spearman’s gene expression correlation based on the average expression of neuropeptides within each cluster. The results were then visualized with a forced embedded layout (Fig. 3C). The separation of the individual clock neuron clusters indicates that differential neuropeptide expression defines very successfully these clusters but much less well the poorly separated DAN clusters. This conclusion may simply reflect the many more neuropeptides in clock neurons than in DANs (Fig. 3B).

### Neural connectivity molecules best identify DANs and clock neurons

**Fig. 4.**
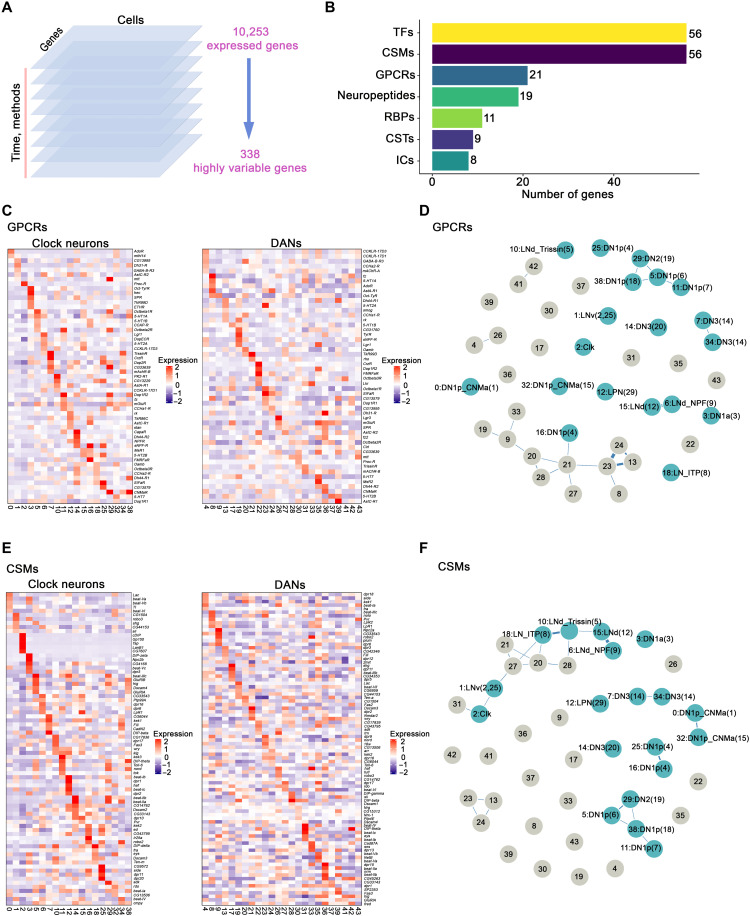
GPCRs and CSMs expression in clock neurons and DANs. (**A**) Schematic workflow of data integration and clustering analysis. We first separated the single-cell gene expression results by time points and methods. In each dataset, 3000 highly variable genes were calculated and only the conserved variable genes (338 genes) were used for final single-cell clustering. (**B**) Bar plot showing the number of highly variable genes from different gene groups including TFs, CSMs, GPCRs, neuropeptides, RNA binding proteins (RBPs), chemical synaptic transmission–related genes (CSTs) and ion channels (ICs). (**C** and **E**) Heatmaps showing the expression levels of GPCRs (C) and CSMs (E) in clock neurons and DANs. (**D** and **F**) Gene expression correlation of GPCRs (D) and CSMs (F) in clock neurons and DANs. We calculated the Spearman’s correlation coefficients between expression patterns of GPCRs (D) and CSMs (F) across different clock neuron and DANs cell types; the result is visualized in a force-embedded layout. Blue nodes represent clock neuron clusters, and gray nodes represent DAN clusters. Each cluster is represented by a node with the width of the edge representing the strength of the gene expression.

To identify other genes that might underlie the cell type definition of DANs and clock neurons, we turned to the highly variable genes that were used during our data integration and clustering analysis. We computed 3000 highly variable genes based on the different raw gene expression datasets separated by time points and methods, of which 338 were expressed and variable in all datasets and then used for further downstream analysis (Fig. 4A). Each cluster expresses a unique combination of these highly variable genes, many of which have been identified as important regulators of behavior and/or physiology (fig. S8), for example, *tup* encodes a TF, which regulates neuronal subtype identity, including motor, serotonergic, and DANs (*44*). Some genes, including *Dh31* and *VGlut* (mentioned above) as well as *DIP-beta*, appear to be cell type defining both in DANs and clock neurons (fig. S8). We next cataloged these highly variable genes into different functional groups (Fig. 4B): The top 3 are TFs, CSMs and G protein–coupled receptor (GPCRs).

The spatial regulation of TF expression is prominent in clock neurons and may contribute to the robust temporal oscillation of many transcripts (*19*). Each DAN cluster also expresses a specific combination of TF genes, very similar to the observed patterns in clock neurons (fig. S9A). However, the gene expression correlation analysis indicates that TFs do not separate the DANs and the clock neurons (fig. S9B), and the clock neurons appear somewhat less well defined by TFs than by neuropeptides: The clock neuron clusters are closer together and have more links in fig. S9B than in Fig. 3C.

GPCRs are another category of neuron connectivity molecules in the highly variable genes (Fig. 4B). GPCRs are 7-transmembrane receptors that interact with many different stimuli including neurotransmitters and neuropeptides and play an important role in the physiology and function of neurons (*45*). More than two-thirds of the 124 GPCR genes encoded by the *Drosophila* genome are expressed in adult clock neurons, and these molecules alone can define clock neuron cell type (*46*). The data here indicate that these transcripts can also define DAN cell type (Fig. 4, C and D).

The highly variable genes contain an almost identical number of CSMs as TFs (Fig. 4B). Although CSMs are critical in mediating interactions between cells in the developing nervous system (*47*), these molecules do not have a comparably well-defined role in the adult nervous system. However, the CSM heatmap indicates impressive cluster-specific expression, in adult DANs and in adult clock neurons (Fig. 4E). Moreover, the gene expression correlation analysis indicates that all single-cell clusters in both populations are very well separated by CSM gene expression (Fig. 4F). Notably, this class of molecules includes the Dpr (defective proboscis extension response) and DIP (Dpr-interacting protein) protein families. Each clock neuron and DAN cluster expresses a specific combination of *dpr* and *DIP* genes (fig. S10, A and B), similar to previous findings on clock neurons (*19*).

**Fig. 5.**
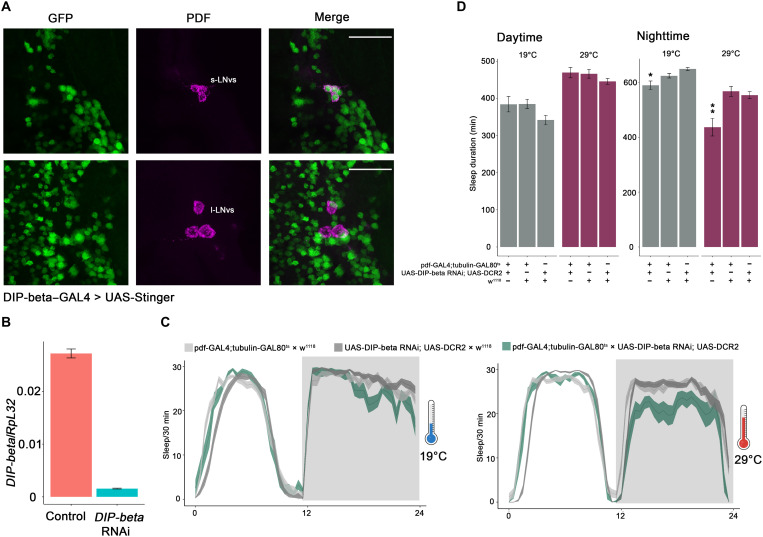
DIP-beta expression in s-LNvs promotes nighttime sleep. (**A**) Confocal stack of images showing antibody staining for GFP (left), PDF (middle), and the merged image (right) in DIP-beta–GAL4 > UAS-Stinger fly brains. Seven-day-old flies were used for the immunohistochemistry. Scale bars, 30 μm. (**B**) Quantitative RT-PCR showing the efficiency of *DIP-beta* RNAi. The RNAi line was crossed with nSyb-GAL4; UAS-EGFP, about 1000 neurons from the control and experimental groups were collected by FACS machine. The cDNA was generated by Smart-Seq3 protocol before the quantitative RT-PCR. The error bars represent SEM. (**C**) Sleep plots of pdf-GAL4; tubulin-GAL80^ts^–driven *DIP-beta* RNAi line (blue) and controls (gray). The flies were raised at 18° before the baseline sleep was recorded at 19° and 29°. The solid lines represent the averaged sleep amount, and the shading represents SEM for each time point. (**D**) Quantification of total sleep duration of pdf-GAL4; tubulin-GAL80^ts^–driven *DIP-beta* RNAi line and controls during the daytime and nighttime. Data are represented as means ± SEM. *P < 0.05 and **P < 0.01 by one-way analysis of variance (ANOVA) test.

To begin addressing the function of these molecules in the adult central brain, we focused on *DIP-beta*. Profiling experiments indicate that its transcript is highly enriched in adult small LNv (s-LNv) clock neurons (*19*). Consistent with this observation, DIP-beta–GAL4 strongly expresses in adult (7-day) s-LNvs (clock neuron s-LNvs) but rarely and then only weakly in large LNvs (clock neuron l-LNvs; Fig. 5A). To address function, we used the only available RNA interference (RNAi) line against *DIP-beta*. Quantitative reverse transcription polymerase chain reaction (RT-PCR) from sorted neurons indicates that this RNAi line reduces *DIP-beta* mRNA expression by more than 90% (Fig. 5B). We then used this RNAi line and pdf-GAL4 along with the classic tubulin-GAL80^ts^ system to knock down *DIP-beta* levels in s-LNvs during adulthood. As the l-LNvs express little or no *DIP-beta*, RNAi knockdown of DIP-beta using pdf-GAL4 should primarily affect s-LNv function. As there is substantially reduced nighttime sleep in this line at the nonpermissive temperature (when GAL80 is inactive), *DIP-beta* likely functions within adult s-LNvs to help these neurons promote nighttime sleep (Fig. 5, C and D). In summary, the data here indicate that neuronal connectivity molecules—neuropeptides (for clock neurons), CSMs, and GPCRs—constitute important classes of neuronal identification molecules in the adult fly nervous system, at least for clock neurons and DANs.

## DISCUSSION

There are about 75 *Drosophila* clock neurons on each side of the adult fly brain. They are defined by their common features, most notably cycling circadian gene expression. They had been divided into small subgroups based on their distinctive anatomical locations and projection patterns, and some had also been distinguished on the basis of immunohistochemistry, namely, the expression of specific neuropeptides and neuropeptide function. However, the advent of single-cell RNA sequencing and the ability to comprehensively profile gene expression have been game changers for neuron characterization (*48*, *49*); our profiling of fly clock neurons with CEL-seq2 increased by approximately a factor of 2, the number of clock neuron subgroups compared to previous definitions. Other features were also notable, especially the spatial and temporal regulation of cell surface proteins (*19*).

To bring some perspective to our previous clock neuron characterization, we assayed here DANs. They were chosen because they are about as numerous as clock neurons in the adult fly brain and because they are arguably the best studied neurons in fly behavior. We first characterized DANs with CEL-seq2, identically to our previous clock neuron work. We then profiled together the transcriptomes of 1979 DANs and 2564 clock neurons (4543 high quality single cells in total), purified from a single strain expressing green fluorescent protein (GFP) in DANs and in clock neurons. By assaying the two populations together, batch effects were eliminated; we also used a different sequencing method for this dual assay, 10X Chromium. This strategy allowed our previous detailed characterization of clock neurons with CEL-seq2 to serve as a benchmark against which the DAN data as well as the new clock neuron data and the 10X sequencing approach could be compared. The 10X sequencing performed very well compared to CEL-seq2: Expressed genes per cell were almost identical, and CEL-seq2 had only about twice as many transcripts/gene (fig. S11).

It is reassuring that an unsupervised clustering algorithm identified all 17 previously defined clock neuron groups in the pooled dataset (*19*). There are, however, 19 clock neuron clusters in the new data, because two previous single clusters are now divided into two clusters. As the Clk856-GAL4 driver used for clock gene expression expresses in 45 neurons per hemisphere, 45/17 to 19 clusters are ~2.5 neurons per cluster. Using this complexity as a benchmark, the results show that DAN heterogeneity is nearly identical. The TH-GAL4 dopaminergic driver expresses in 63 DANs per hemisphere, which were categorized into 24 DAN clusters with an average of 63/24 to 2.6 neurons per cluster. Many of these clusters correspond to known DAN subsets, whereas others express discrete marker genes. They should facilitate further anatomical identification and functional studies.

If the numbers are characteristic of all 140 DANs per hemisphere, then there are at least 50 to 60 different DAN transcriptome types per hemisphere. This level of heterogeneity is not very different from the classification of neuron morphology and connectivity based on the hemibrain EM dataset (fig. S12, A and B) (*1*), suggesting that the latter has a gene expression underpinning. In this context, Waddell and colleagues (*50*) have identified 20 PAM-γ5 subtypes by anatomy, and it would be expected if many/most of these anatomical subtypes are also discrete by transcriptional profiling.

A detailed comparison between the DAN and clock neuron heterogeneity data and the hemibrain EM heterogeneity data is difficult. This is because the average numbers from the EM study accommodate very different kinds of heterogeneity and neurons (fig. S12, A and B). Moreover, these numbers are continually being updated. We know, for example, that the seven described categories of circadian neurons are underestimate. For example, recent anatomy and connectome analyses of the six LNd clock neurons indicate that they fall into four different groups based on anatomy rather than one group with six neurons; these four groups precisely match our previous classification based on gene expression (*19*, *51*).

This complexity of the adult fly brain transcriptome including that of CSM expression is not predicted by previous results. Although there is even greater diversity in *Drosophila* visual and olfactory neurons during developmental wiring, it is not maintained in the adult, especially between visual system neurons that only differ in their connectivity and perform very similar functions (*7*, *9*–*12*). The visual system may therefore not be characteristic of most central brain neurons. Moreover, whole fly brain single-cell sequencing efforts identified only a single DAN and a single clock neuron cluster (fig. S13, A to C). The very recent whole fly cell atlas indicates more heterogeneity, but the identified circadian cells are predominantly from photoreceptor, cone, and epithelial cells (fig. S13, D and E) (*4*, *5*, *8*); there is no indication of the known clock neuron complexity within the central brain [e.g., (*19*)]. It is likely that these data are too shallow and specific cells insufficiently numerous to reveal the many clock and dopaminergic central brain neuron groups. Transcriptomic definition of cell type from the adult fly central brain may require at least 1 million cells, many more than what has been done to date in whole brain and whole fly studies (*3*–*5*, *8*). One million cells would be sufficient to profile 30 to 100 copies of the perhaps 10,000 to 30,000 different cell types indicated by the EM connectome of the fly brain. An alternative approach is to use cell or nuclear purification from a very large number of specific drivers as we have done here for two drivers.

It is expected that DANs manifest so few cycling transcripts compared to clock neurons (fig. S5), as DANs probably lack a molecular clock (*31*). In this context, recent in vivo calcium imaging shows that some DAN subgroups exhibit cycling neural activity rhythms, which are apparently driven by upstream clock neurons (*52*, *53*). This might make the small number of DAN cycling transcripts of interest, reflecting perhaps previously unidentified or already known activity-regulated genes (*54*). However, defining this cycling gene expression with certainty is currently challenging when the number of cycling transcripts is so low and the proper criterion for cycling gene expression uncertain (fig. S5).

Both drivers, Clk856-GAL4 and TH-GAL4, fail to express in a substantial fraction of their expected patterns, presumably because they are simply missing important regulatory information that normally dictates clock or pale (TH) expression. The Clk856-GAL4 driver expresses in only a few DN3 clock neurons despite expressing in all clock neurons with well-characterized functions. The enigmatic DN3s constitute about half of the clock neuron population, 30 to 40 cells per hemisphere. The TH-Gal4 driver also fails to express in about half of the fly brain DANs, about 78 of 141 neurons per hemisphere. These missing DANs include most of the numerous PAM DANs, i.e., TH-GAL4 expresses in only 13 PAMs per hemisphere rather than the known 100 PAMs per hemisphere. These missing neurons may account for some of the data shortcomings and the few differences between the clock neuron and DAN populations. For example, there is much more neuropeptide transcript expression in clock neurons than in DANs (Fig. 3). This is presumably why neuropeptide expression can define clock neuron clusters much more successfully than DAN clusters (Fig. 3C). This distinction may be less notable if DN3 clock neurons express relatively few neuropeptide transcripts compared to Clk856-GAL4–positive clock neurons and/or if the missing DANs express many neuropeptide transcripts compared to the DANs identified by TH-GAL4. In any case, it is uncertain whether the current characterization of neuropeptide expression in clock neurons or in DANs reflects the general case for the fly brain if, indeed, there is one. It is also uncertain whether the mammalian suprachiasmatic nucleus is similar to fly brain clock neurons, namely, an apparently richer source of neuropeptide expression than elsewhere in the mammalian brain (*55*).

TFs also separate clock neuron clusters more successfully than DAN clusters (fig. S9B). In contrast to neuropeptide transcript expression, however, there is no indication that TF expression is different between DANs and clock neurons; the heatmaps appear similar, and the relevant GO term is even more highly ranked in DANs than in clock neurons. Perhaps DAN definition is more dependent on TF expression during development than clock neurons definition.

We were surprised to find that neuron connectivity molecule transcripts are the best definers of neuron identity for DANs and for clock neurons. CSMs are even superior to TFs for clock neuron definition (compare Fig. 4E with fig. S9). We had previously found that GPCRs can identify clock neurons (*46*), and these data extend this conclusion to DANs. This ability to define adult neuron subtype is particularly notable in the case of neuropeptides and GPCRs. This is because there are relatively few of these genes in the set of 338 highly variable genes that are used to cluster the cells (Fig. 4B), many fewer than the number of CSM genes. The success of GPCRs recalls the area code hypothesis, a transmembrane receptor cell surface code for embryo assembly (*56*), as well as the success of neuropeptides and GPCR pairs in cortical neuron definition (*57*).

Neuropeptides define very well only clock neurons, reflecting no doubt the relative abundance of specific neuropeptide gene expression in clock neurons, whereas GPCRs do equally well in defining DANs and clock neurons. It is therefore tempting to speculate that there is more intraclock neuron circuit communication between neuropeptides and GPCRs, as known for PDF and PDF receptor, whereas DAN GPCRs are stimulated by ligands that come principally from non-DAN sources.

CSMs are critical for nervous system wiring during development, best illustrated by their remarkably complex expression patterns during fly visual system development. As an example, the Dpr proteins have affinity for specific partner DIP proteins, which together help drive synapse specificity during visual system development (*47*, *58*). These adult data suggest that CSMs are also important for wiring specificity and/or synaptic strength in the adult brain. Perhaps plasticity, responding to different environments, is also dependent on these molecules.

As a first test of the hypothesis that CSM expression is important in the adult, knocking down *DIP-beta* expression in adult ventral lateral neurons (LNv) neurons results in substantially reduced nighttime sleep (Fig. 5). This is consistent with the early night peak expression of *DIP-beta* in small ventral lateral neurons (s-LNvs) (*19*) and suggests that CSMs play a prominent role in adults same as they do during the development. It is known that LNv neuronal activity regulates the total amount of sleep and the rate of sleep onset (*59*). The LNv neuropeptide PDF is activity promoting, but the s-LNvs also express short neuropeptide F (sNPF), which is sleep promoting (*59*, *60*). As a recent study indicates unexpectedly complex regulation of neuropeptide release from s-LNvs (*61*), *DIP-beta* knockdown may interfere with the proper interaction of LNvs with downstream partner molecules and neurons and thereby affect normal neuropeptide function from s-LNvs and as a consequence nighttime sleep level. Although these observations need to be extended to other molecules, to morphology, to DANs, and eventually to additional classes of adult neurons, our findings emphasize the importance and broad reach of neuron connectivity molecules. They extend from anatomy and brain wiring to specific gene expression patterns and even now to the behavioral repertoire of individual adult brain neurons.

## MATERIALS AND METHODS

### Fly strains and rearing

Flies were reared on standard cornmeal medium with yeast under 12:12 LD conditions at room temperature. The fly lines used in this study are listed in table S1. Equal numbers of males and females were used in all the single-cell RNA sequencing library preparation.

### Fluorescence-activated cell sorting

We used enhanced GFP (EGFP) to label the targeted neurons. Flies were entrained in 12:12 LD cycles at 25°C conditions for 3 days before dissection. For CEL-seq2 experiments, time points were taken every 4 hours within a day, and ZT02 (2 hours after lights on) and ZT14 were used for the 10X Chromium experiment. Fly brains were dissected in cold dissection saline [9.9 mM Hepes-KOH (pH 7.4), 137 mM NaCl, 5.4 mM KCl, 0.17 mM NaH_2_PO_4_, 0.22 mM KH_2_PO_4_, 3.3 mM glucose, and 43.8 mM sucrose] with neuronal activity inhibitors (20 μM 6,7-dinitroquinoxaline-2,3-dione, 0.1 μM tetrodoxin, and 50 μM d,l-2-amino-5-phosphonovaleric acid). The brains were digested with papain (50 U/ml, ~2 μl per brain; Worthington Biochemical, #LK003176) at room temperature for 30 min. Brains were then resuspended and washed twice with ice-cold active Schneider's *Drosophila* Medium (SM) medium after the digestion. To get the single-cell suspension, we used flame-rounded 1000-μl pipette tips with different sized openings and triturated the brains until most of the tissues were dissociated. The resulting cell suspension was filtered by a 100-μm sieve. Hoechst dye (one drop per 0.5 ml of sample; Invitrogen, #R37605) was added into the sample tube to stain the nucleus before the single-cell sorting. A BD Melody fluorescence-activated cell sorting (FACS) machine in single-cell sorting mode was used for cell collection. Only the GFP- and Hoechst-positive single cells were collected. The collection devices were kept at 5°C constantly during the sorting process.

### Single-cell RNA library preparation by modified CEL-seq2

We used our previous modified CEL-seq2 method for the single-cell RNA sequencing of DANs at multiple time points. Briefly, single DANs were first sorted into 384-well plates prefilled with 0.6 μl of primer mix (dNTP and primers), and plates with sorted cells were centrifuged at 3000*g* for 1 min at 4°C and then stored in −80°C until further processing. There are 96 poly-T tailed primers in our method with which we can make four libraries from a 384-well plate. To increase the throughput, we used an Eppendorf epMotion liquid handler to dispense first-strand synthesis reagents and second-strand synthesis reagents mixes. cDNA from the same primer set was pooled together and cleaned by 0.8-fold AMPure beads before the in vitro transcription (overnight). Antisense RNA was converted to double-stranded DNA with a random primer and T7-RA5 primer. The resulting cDNA underwent another final second round in vitro transcription (IVT) step at 37°C overnight was followed by ExoSAP treatment (Affymetrix 78200) for 15 min at 37°C. Other steps were performed as described in the CEL-seq2 protocol.

### Single-cell RNA library preparation by 10X Chromium

The same method was used to make the single-cell suspension as described above. We first collected GFP- and Hoechst-positive single cells in a 1.5-ml Eppendorf tube with 0.3 ml of collection buffer [phosphate-buffered saline (PBS) + 0.04% bovine serum albumin]. The cells were spun down on a centrifuge by 700*g* for 10 min. We used the Chromium Single Cell 3′ Kit (v3) of 10X Genomics. The libraries were prepared according to the standard user guide (CG000315 Rev. B) from 10X without any modifications.

### Library sequencing and raw data processing

Both the CEL-seq2 libraries and 10X libraries were sequenced by Illumina NextSeq 500 with the High Output Kit v2.5 (75 cycles). zUMIs and Cell Ranger were used to map the sequencing data to the *Drosophila* genome (dm6) and count the reads from CEL-seq2 and 10X Chromium separately (*33*). Only the alignments to annotated exons were used for UMI quantitation.

The sequencing depth in CEL-seq2 and 10X are different, so we used two different criteria to filter out low-quality cells in CEL-seq2 and 10X experiments before the clustering analysis. For the cells in CEL-seq2 experiment, we used the following criteria: (i) fewer than 500 or more than 6000 detected genes (where each gene had to have at least one UMI aligned); (ii) fewer than 4000 or more than 75000 total UMI; and (ii) gene expression entropy smaller than 5.0, where entropy was defined as −nUMI x* ln*(nUMI) for genes with nUMI > 0, where nUMI was a number of UMI in a cell. For the cells in 10X experiment, we used the following criteria: (i) fewer than 300 or more than 3500 detected genes (where each gene had to have at least one UMI aligned), (ii) fewer than 1000 or more than 55,000 total UMI, and (iii) gene expression entropy smaller than 5.0. We used Scrublet to detect the possible doublets in the 10X experiment; these cells were excluded from the following analysis.

### Dimensionality reduction and clustering

The method used for single-cell clustering has been described previously (*19*). Briefly, we integrated the cells from different methods and time points using integration functions from the Seurat (version 3.0.2) package (*62*). First, we separated the single-cell data by methods and time points and used the SCTransform function to transform data using the normalization and variance stabilization of counts. The batch effect was removed by regressing out numbers of genes, UMIs, detected genes per cell, sequencing batches, and percentage of mitochondrial transcripts. We computed 3000 variable genes at each time point and method and found a subset of variable genes that were common to eight conditions (six time points from CEL-seq2 and two time points from 10X). From this set of common variable genes, we removed the mitochondrial, ribosomal, and transfer RNA genes. The resulting genes were used for integrating data using Seurat *FindIntegrationAnchors* and *IntegrateData* functions. Last, we performed principal components analysis (PCA) on scaled gene expression vectors (*z* scores) and reduced the data to the top 49 PCA components. This analysis resulted in 70 initial clusters, we next filtered the clusters on the basis of the following criterion: First, all clusters must have cells from CEL-seq2 and 10X; second, among the CEL-seq2 data in each cluster, there should be cells from all time points throughout the day; last, clusters with low number of genes and transcripts were excluded. The cells in confident clusters were iterated one more time for the clustering as described above. We visualized the data using *t*-distributed stochastic neighbor embedding (t-SNE) except where indicated specifically and reported a relative, normalized number of UMIs in a cell as TP10K (transcripts per 10 thousand transcripts).

### Differentially expressed genes in each cluster

The Seurat *FindAllMarkers* function with a negative binomial generalized linear model was used to identify the differentially expressed in each cluster. The *P* values were adjusted for multiple hypothesis testing using Bonferroni method. We used an adjusted *P* value significance of 0.05 and fold change cutoff of 1.25 as the threshold of significant differential expression.

### Matching single-cell and bulk RNA sequencing in DANs

The bulk RNA sequencing results from different DAN subgroups were downloaded from (*41*). Only the results from FACS-sorted samples were included in the current study. We first computed the enriched marker genes in DAN clusters by *FindAllMarkers* function from Seurat. The top 50 enriched genes from each cluster were used to compute the gene expression correlation between single-cell and bulk RNA sequencing result.

### Immunohistochemistry

Immunohistochemistry was performed on 3- to 7-day-old flies. Flies were fixed with 4% (v/v) paraformaldehyde with 0.5% Triton X-100 for 2 hours and 40 min at room temperature. Brains were dissected in phosphate-buffered saline with 0.5%Tween 20 (PBST) and then washed twice (10 min) in 0.5% PBST buffer with rotation. The 10% normal goat serum (Jackson ImmunoResearch) was used for blocking overnight at 4°C. Mouse anti-TH at 1:1000 dilution, rat anti-TIM at 1:200 dilution, and chicken anti-GFP antibody at a 1:1000 were used as primary antibody and incubated with the brains overnight at 4°C, and the brains were then washed twice (10 min) in 0.5% PBST buffer at room temperature. The corresponding secondary antibodies were added and incubated overnight at 4°C. Brains were mounted in VECTASHIELD (Thermo Fisher Scientific) and imaged on a Leica SP5 confocal microscope. The images were processed by ImageJ.

### Cycling transcripts analysis

JTK_CYCLE and Lomb-scargle (LS) methods from MetaCycle package were used for cycling transcripts analysis. The genes with an expression higher than 0.5 TP10K in each cluster were used for cycling analysis. Two different cutoffs were used to call a cycler: cycling amplitude (maximum expression divided by minimum expression) of at least 1.5-fold, a maximal expression of at least 0.5 TP10K, JTK cycle, and LS *P* values of less than 0.05 (loose criteria) or JTK cycle and LS Benjamini-Hochberg–corrected *q* value of less than 0.05 (stringent criteria).

### Behavior analysis

The flies were crossed and raised at 18°. Five-to 7-day-old male flies were used for the behavioral experiments and locomotor activity, and sleep of individual flies was measured by *Drosophila* activity monitors (Trikinetics Inc.), in which individual flies were placed into glass tubes with food (2% agar and 4% sucrose) on one end and a plug to close the tube on the other end. The flies were entrained under 12:12 LD conditions for at least 3 days. Each experiment was performed twice and got similar results. The activity and sleep analysis were performed with MATLAB. Statistical analysis was performed using a one-way analysis of variance (ANOVA) (www.statskingdom.com), and *P* < 0.05 compared to all control groups was considered significant.

## Supplementary Materials

### This PDF file includes:

Figs. S1 to S13 Table S1 References

## Notes

View/request a protocol for this paper from *Bio-protocol*.
