# Supplementary material for: Neural connectivity molecules best identify the heterogeneous clock and dopaminergic cell types in the Drosophila adult brain
Source: Sci Adv. 2023 Feb 22;9(8):eade8500. doi: 10.1126/sciadv.ade8500 (PMC9946362; doi:10.1126/sciadv.ade8500)
Supplement: Supplementary file 1 — Figs. S1 to S13 Table S1 References [file sciadv.ade8500_sm.pdf]

Science Advances
AAAS

# Supplementary Materials for

## Neural connectivity molecules best identify the heterogeneous clock and dopaminergic cell types in the *Drosophila* adult brain

Dingbang Ma *et al.*

Corresponding author: Michael Rosbash, rosbash@brandeis.edu

*Sci. Adv.* **9**, eade8500 (2023)
DOI: 10.1126/sciadv.ade8500

**The PDF file includes:**

Figs. S1 to S13
Table S1
Legend for table S2
References

**Other Supplementary Material for this manuscript includes the following:**

Table S2

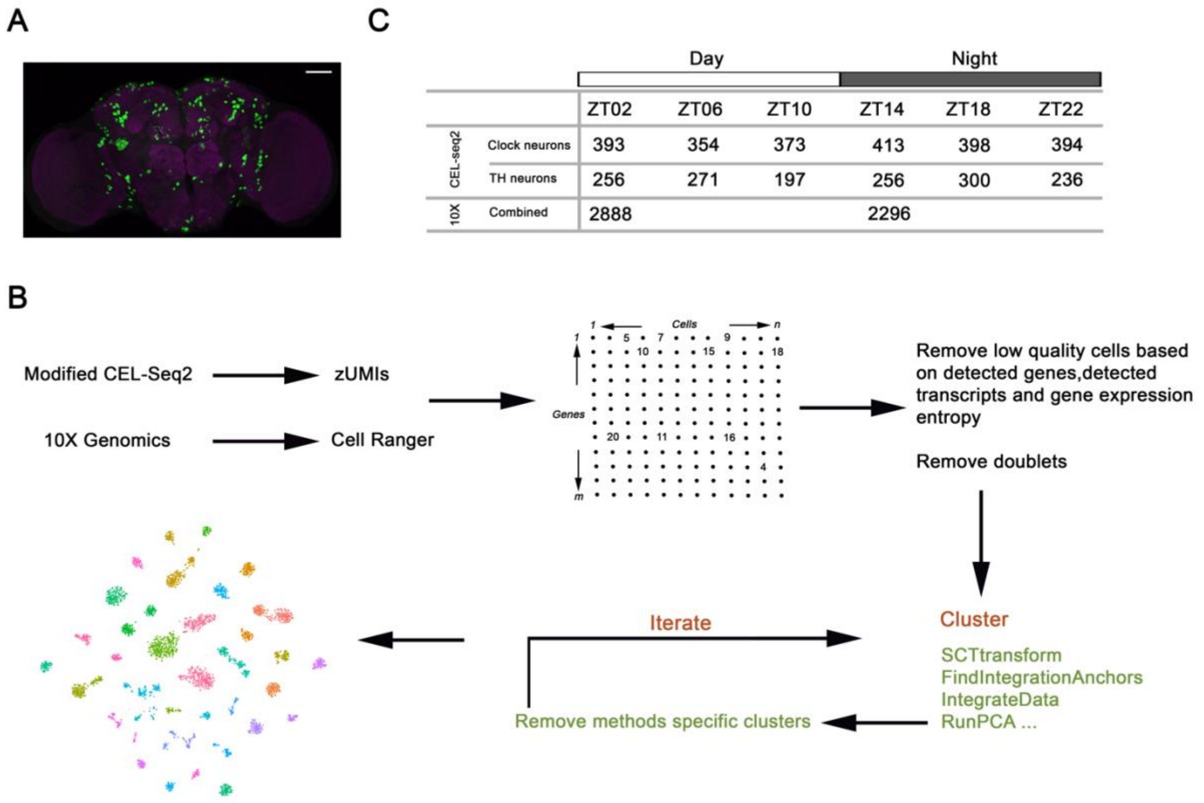


**Fig. S1. Integration of CEL-Seq2 and 10X Chromium datasets.** (A) Expression pattern of *Clk856-GAL4;TH-GAL4 > UAS-Stinger-GFP* co-stained with anti-GFP (green) and nc82 (magenta). Scale bar is 50 µm. (B) Flow diagram showing the data processing. zUMIs and Cellranger were used to map and count single cell RNA libraries from CEL-Seq2 and 10X Chromium separately. The cells were filtered based on the number of detected genes, transcripts and gene expression entropy. Scrublet was used to identify possible doublets in 10X Chromium data; these doublets were excluded in the downstream analysis. (C) The number of high-quality cells from CEL-seq2 and 10X Chromium at each time point after initial filtering.

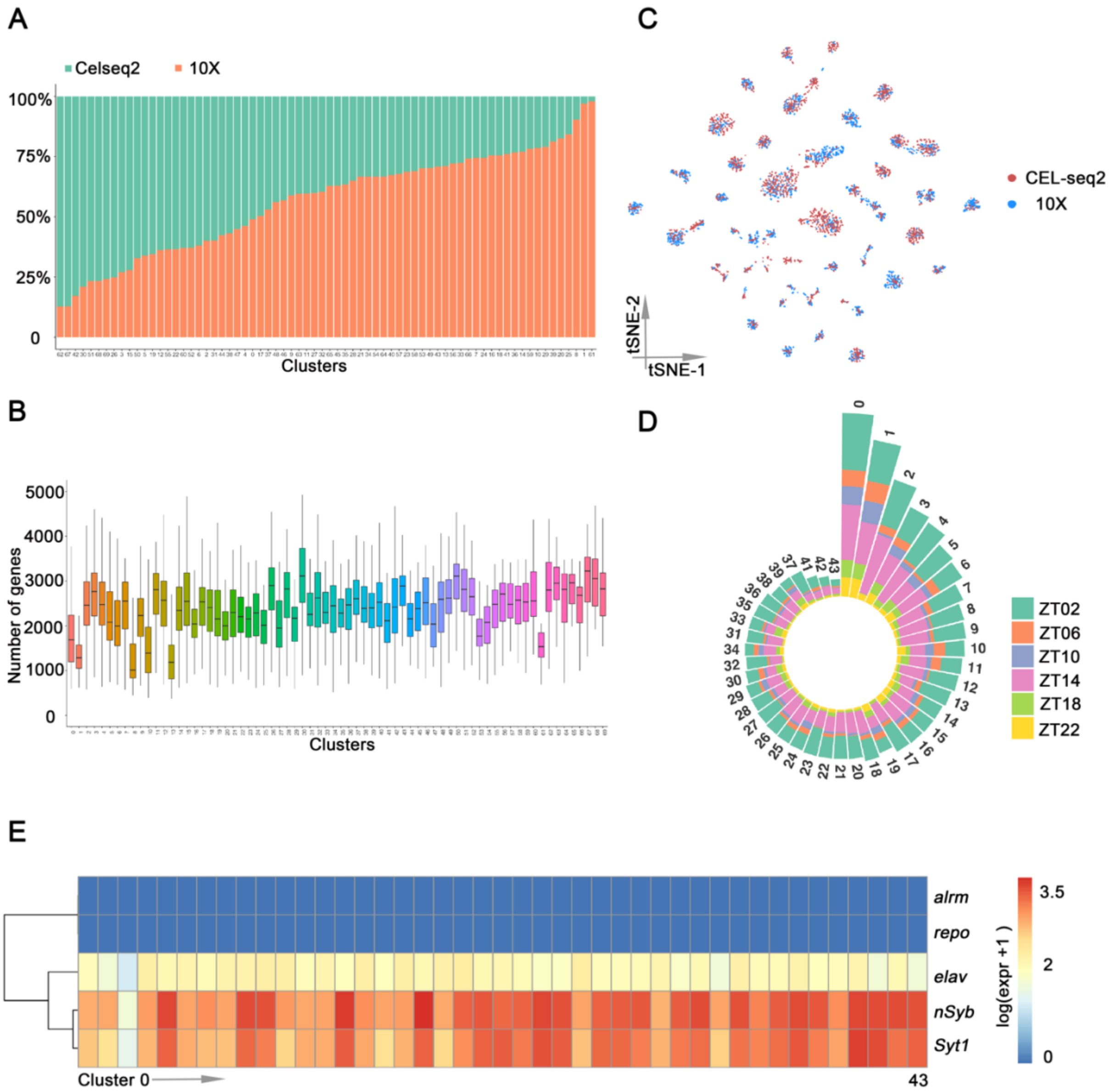


**Fig. S2. Identification of high confidence clusters.** (A) The percentage of cells from CEL-seq2 and 10X Chromium in each cluster. The clusters are ordered by the percentage of cells from the CEL-Seq2 method. (B) Box plot showing the number of detected genes in each cluster. Each cluster has relatively similar numbers of genes with some exceptions. Numbers on the x-axis represent the 70 original clusters. (C) t-SNE plot showing the cells from CEL-seq2 (red) and 10X Chromium (blue) in the final 43 high confidence clusters. (D) Circled bar plot showing that in high confidence clusters there are cells from 6 time points in Light: Dark conditions. (E) Heatmap showing the glial and neuronal marker gene expression in all clusters.

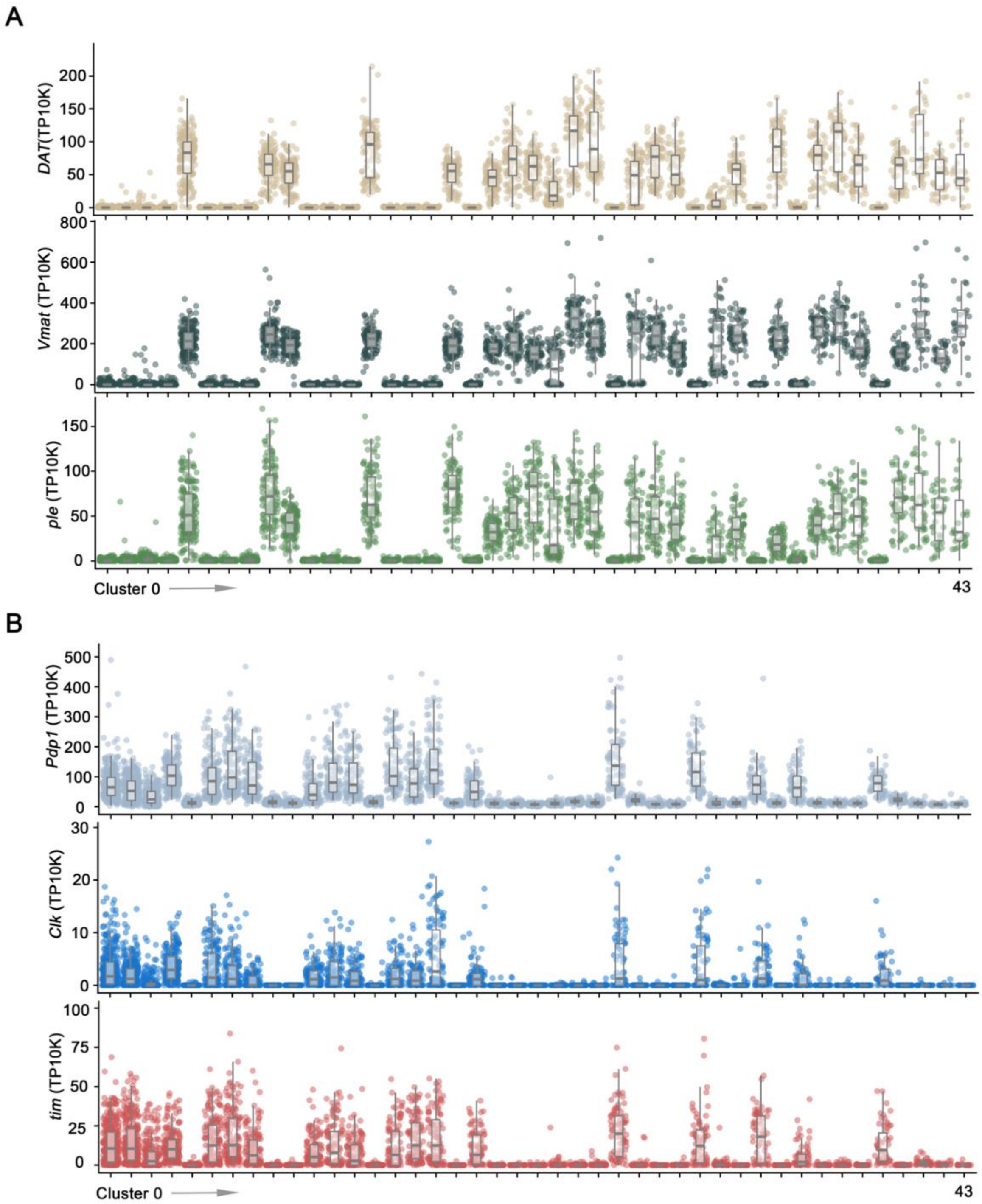


**Fig. S3. Marker gene expression in DAN and clock neuron clusters.** (A-B) Dot plot showing that *ple*, *Vmat* and *DAT* are enriched in DANs (A), *tim*, *Clk* and *Pdp1* are exclusively expressed in clock neurons (B). Gene expression levels for each cell were normalized by total expression level; we report transcripts per 10 thousand transcripts (TP10K). Clusters are ordered by size.

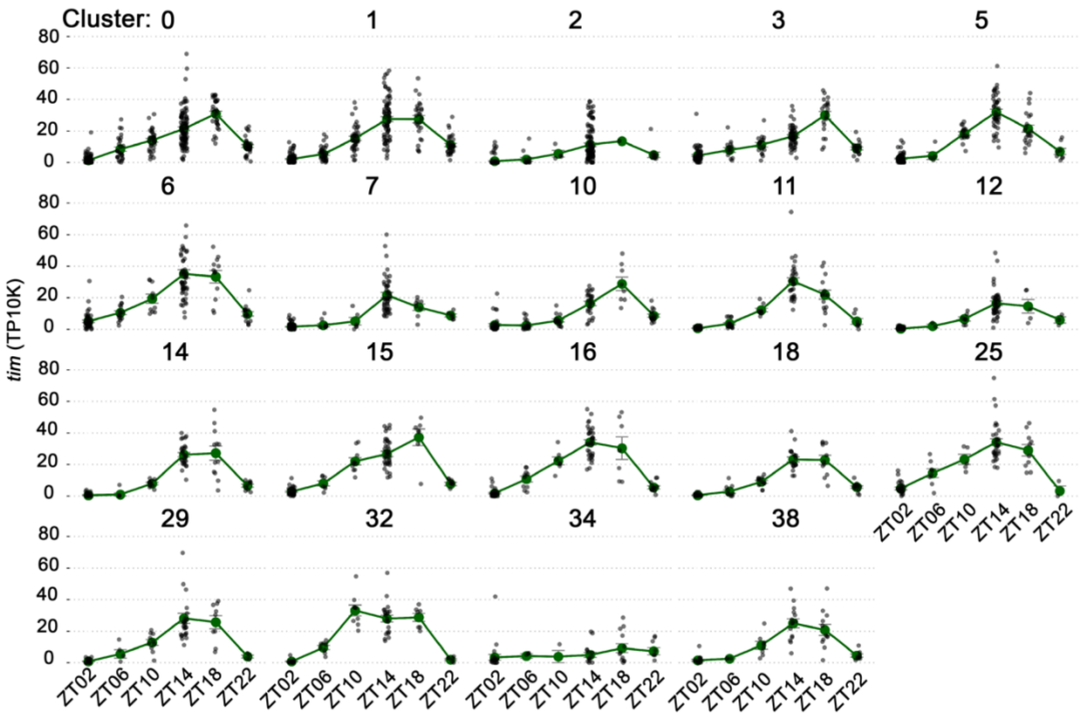


**Fig. S4.** ***tim*** **expression in all clock neuron clusters.** *tim* expression in single cells are shown in gray dots, the green dots represent the average *tim* expression in each cluster at different time points and the error bars represent SEM. Gene expression levels for each cell were normalized by total expression level.

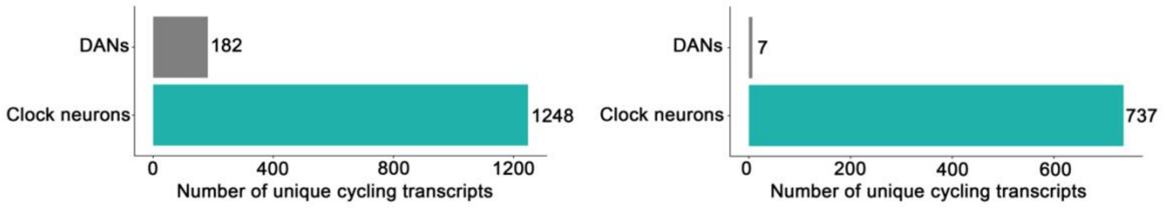


**Fig. S5. Number of cycling transcripts in DAN and clock neurons.** The following cycling cutoffs were used: cycling amplitude (maximum expression divided by minimum expression) of at least 1.5-fold, a maximal expression of at least 0.5 TP10K, JTK cycle and LS p-values of less than 0.05 (left panel) or JTK cycle and LS Benjamini-Hochberg corrected q-value of less than 0.05 (right panel).

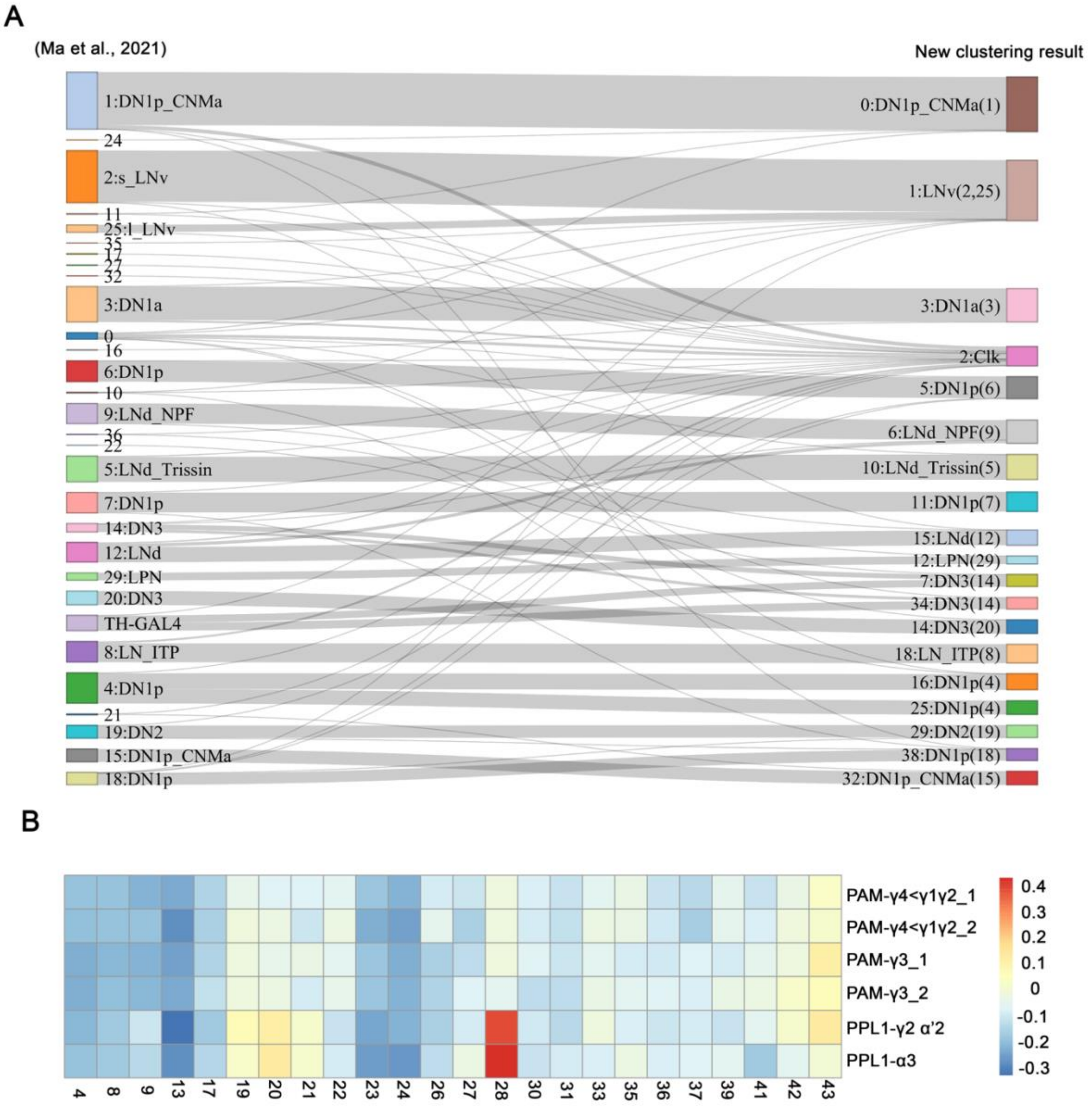


**Fig. S6. Comparison of single cell clusters in the current study with previous results.** (A) Sanky plot showing the contribution of predefined clock neurons clusters (left) to the classification (right) in the current study. Each node represents a single cell cluster. For comparison, each clock neuron cluster retains its original identifying number in the parentheses as it was reported previously. (B) Heatmap showing the gene expression correlation between single cell clusters and different DAN subgroups. Only the transcriptomic results from FACS sorted cells were included in the analysis.

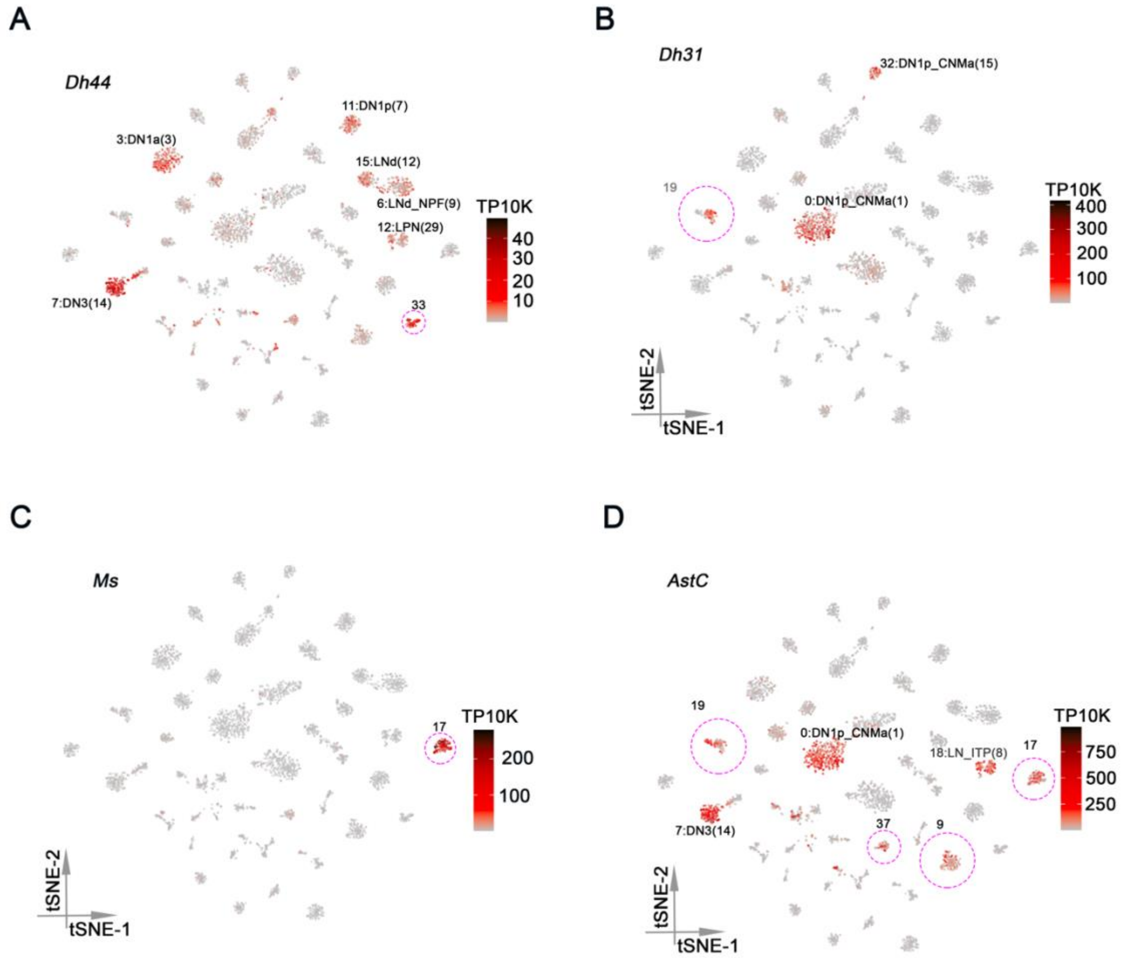


**Fig. S7. Neuropeptide expression in clock neuron and DAN clusters.** (A-D) t-SNE plots showing *Dh44* (A), *Dh31* (B), *Ms* (C) and *AstC* (D) expression in all clusters. Each cell is colored by the expression level with red indicating high expression and gray indicating low expression. The DAN clusters are highlighted by dashed red circles.

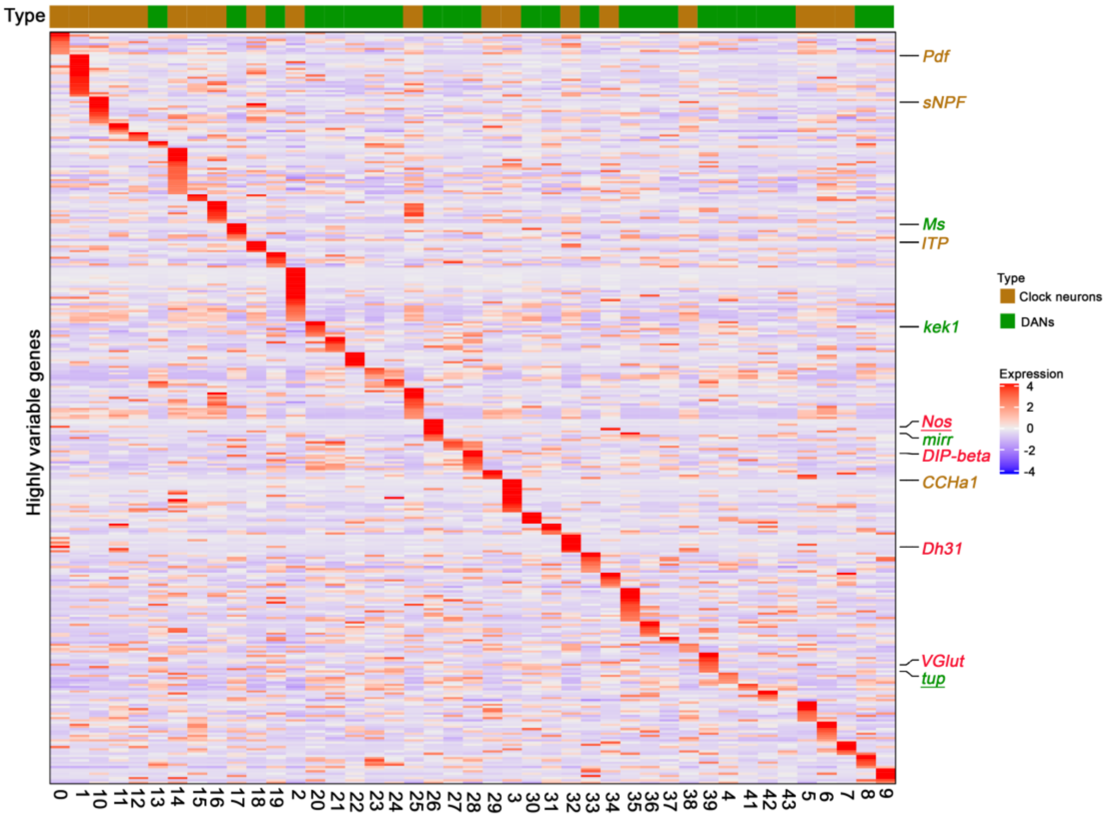


**Fig. S8. Highly variable genes expression in clock and dopaminergic neuron clusters.** Heatmap showing the expression levels of 338 highly variable genes in all 43 clusters. Red indicates high expression and purple indicates low expression. The color bars on the top represent clock and dopaminergic neuron clusters. Representative genes from DANs are labeled in green, the genes from clock neurons are labeled in brown and gene expressed both in DANs and clock neurons are labeled in red. It has been shown that *Nos* and *tup* regulate the physiology and sub-type identity of DANs(*41*, *44*), *kek1* and *mirr* were identified in DANs previously(*4*, *63*).

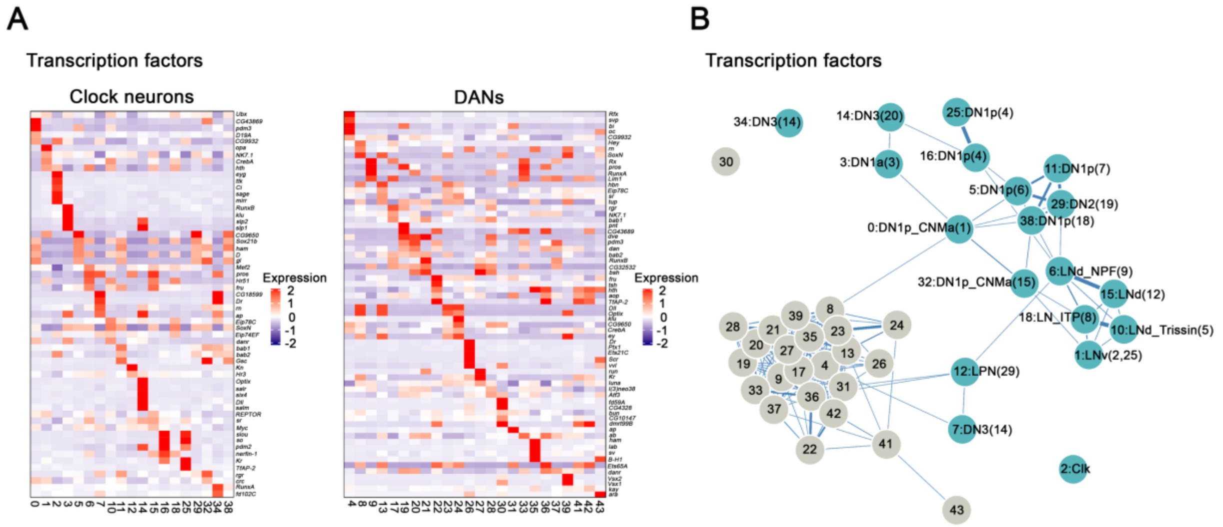


**Fig. S9. Transcription factors expression in identified cell type.** (A) Heatmaps showing the expression levels of transcription factors in clock neurons and DANs. (B) Gene expression correlation of transcription factors in clock neurons and DANs. We calculated the Spearman's correlation coefficients between expression patterns of transcription factors across different clock neuron and DANs cell types; the result is visualized in a force embedded layout. Each cluster is represented by a node with edge width representing the strength of the gene expression. Blue nodes represent clock neuron clusters and gray nodes represent DAN clusters.

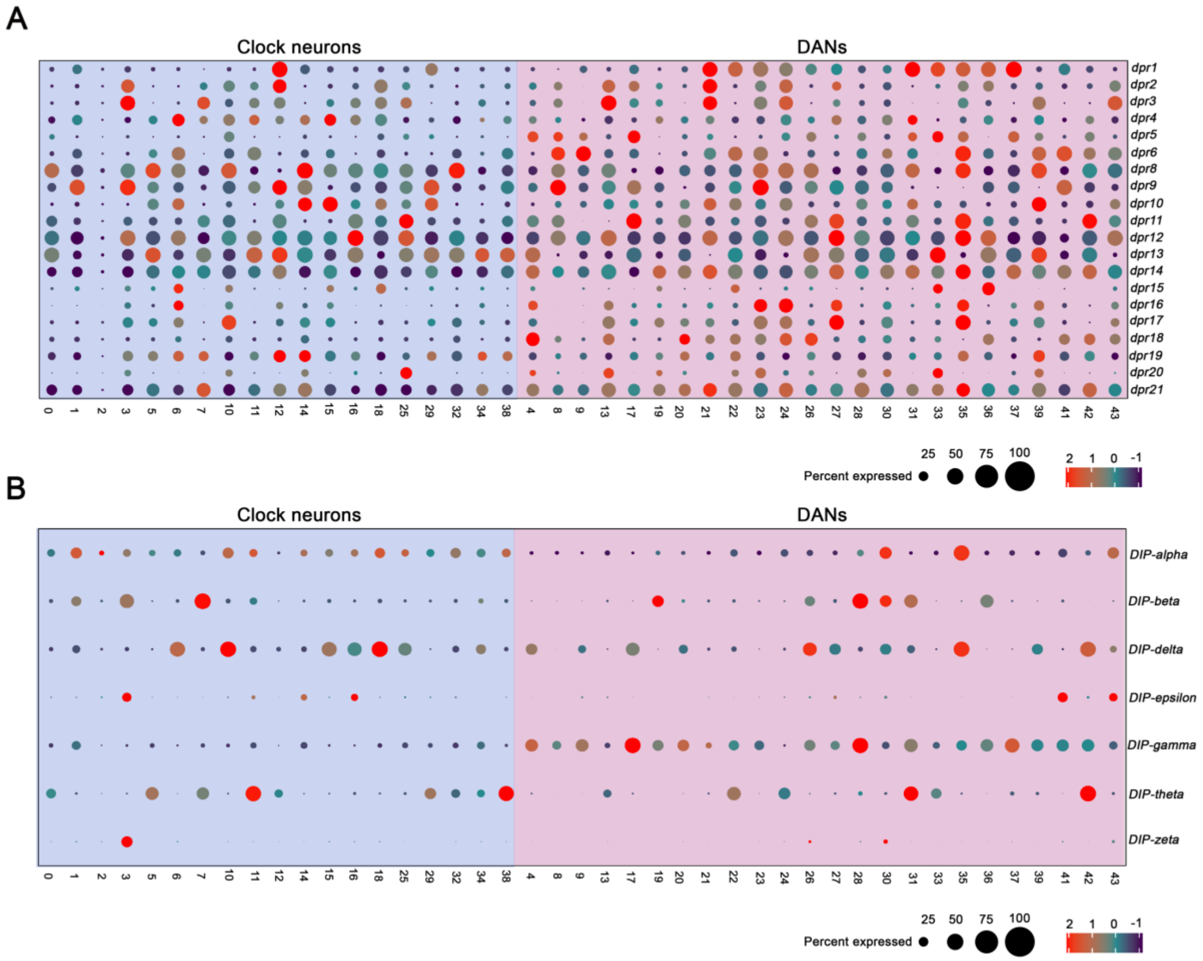


**Fig. S10. DIP and dpr gene expression in identified cell types.** (A-B) Dot plots showing the gene expression of *dpr* (A) and Dpr interacting protein (DIP) (B) members in identified clusters. The size of the dot indicates what percentage of cells in a particular cluster that express the indicated gene. Color indicates the mean expression within that cluster.

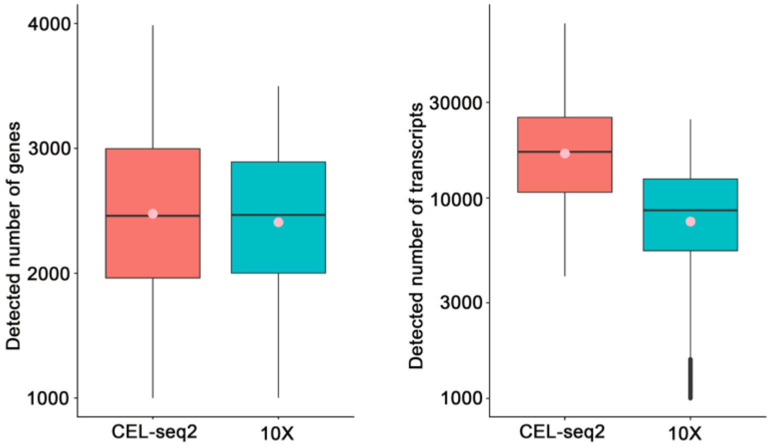


**Fig. S11. Number of detected genes and transcripts by modified CEL-seq2 and 10X.** The number of genes (left) and transcripts (right) in single cells from plate- (red) and droplet-based (blue) methods are plotted. The pink points represent average number of genes and transcripts in these two methods.

**A**

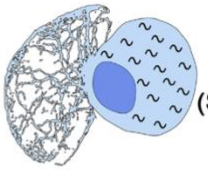


| | Neurons | Labeled | T-types | T/t |
|---|---|---|---|---|
| Clock neurons | 75 | 45 | 19 | ~2.4 |
| DANs | 141 | 63 | 24 | ~2.6 |

**B**

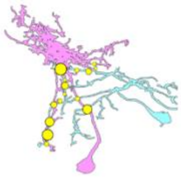


| | Neurons | m-types | c-types | c/t | |
|---|---|---|---|---|---|
| Clock neurons | 27 | 7 | 7 | ~3.9 | |
| DANs | 335 | 35 | 43 | ~7.8 | Including MB associated DANs |
| | 23 | 14 | 14 | ~1.7 | Excluding MB associated DANs |

Scheffer et al., (1)

**Fig. S12. Identified transcriptomic and anatomical clusters in clock neurons and DANs.** (A) A summary of identified gene expression clusters (T/t, average number of cells per transcriptomic type which are based on gene expression similarity) in the current study. (B) Cell types identified by morphology and connectivity (c/t, average number of cells per connectivity type) from the hemi-brain EM dataset, m-types are the number of morphology types. The cell number includes some neurons on the contralateral side, they represent the number of cells that are included in the clustering, but not the number of neurons per brain side (*1*).

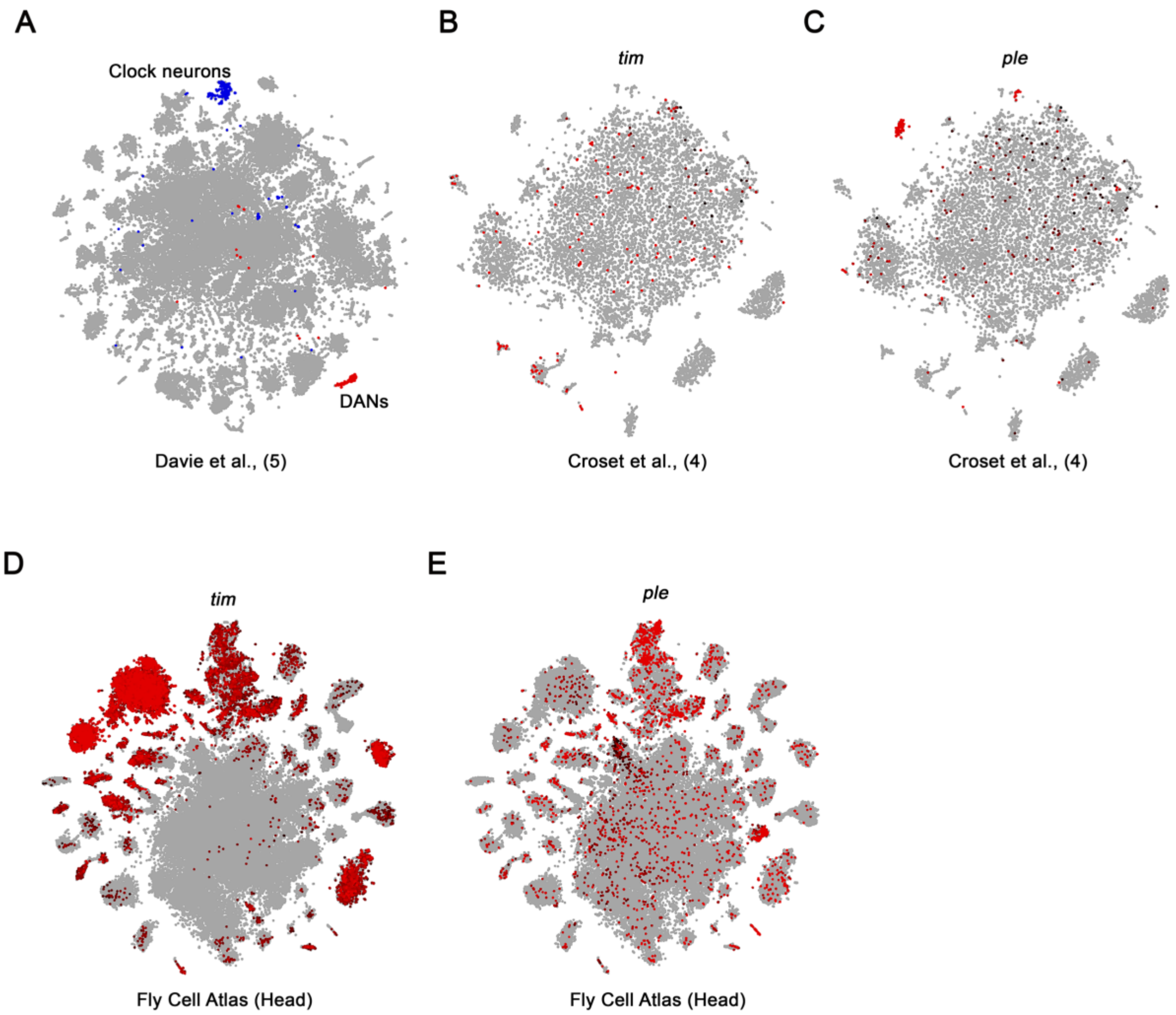


**Fig.S13. DAN and clock neuron clusters identified in previous results.** (A) t-SNE plots showing the identified clock neuron (blue) and DAN (red) clusters from (*5*). (B-C) *tim* (B) and *ple* (C) expression in (*4*). (D-E) *tim* (D) and *ple* (E) expression in Fly Cell Atlas head result (*8*).

## Table S1. Key resources

| Reagent types | Designation | Source or reference | Identifiers | Additional information |
|---|---|---|---|---|
| Genetic reagent (*D. melanogaster*) | UAS-Stinger | BDSC | RRID:BDSC_84277 | |
| Genetic reagent (*D. melanogaster*) | Clk856-GAL4 | (*30*) | Flybase: FBtp0069616 | |
| Genetic reagent (*D. melanogaster*) | UAS-EGFP | BDSC | RRID:BDSC_5428 | |
| Genetic reagent (*D. melanogaster*) | *DIP-Beta-GAL4* | Provided by Zinn lab | | |
| Genetic reagent (*D. melanogaster*) | *DIP*-beta *RNAi* Dmel\P{TRiP.HMS01774}attP40 | Provided by Zinn lab | | |
| Genetic reagent (*D. melanogaster*) | pdf-GAL4 | (*64*) | | |
| Genetic reagent (*D. melanogaster*) | *tubulin*-GAL80$^{ts}$ | BDSC | BDSC_7018 | |
| Genetic reagent (*D. melanogaster*) | UAS-FRT-STOP-FRT-CsChrimson. mVenus and LexAop-FLP | (*65*) | RRID:BDSC_84277 | |
| Genetic reagent (*D. melanogaster*) | VGlut-LexA | (*66*) | | |
| Genetic reagent (*D. melanogaster*) | TH-GAL4 | (*31*) | | |
| Antibody | Anti-TH Mouse monoclonal | IMMUNOSTAR | | 1:1000 |
| Antibody | Anti-TIM Rat monoclonal | Laboratory of Michael Rosbash | RRID: AB_2753140 | 1:200 |
| Antibody | Anti-PDF Mouse monoclonal | Developmental Studies Hybridoma Bank | AB_760350 | 1:500 |
| Antibody | anti-GFP Chicken | Abcam | RRID: AB_300798 | 1:1000 |
| Antibody | Goat anti-mouse polyclonal | ThermoFisher | RRID: AB_2536185 | 1:200 |

| Reagent types | Designation | Source or reference | Identifiers | Additional information |
|---|---|---|---|---|
| Antibody | Goat anti-Chicken polyclonal | ThermoFisher | AB_2534096 | 1:200 |
| Software, algorithm | FIJI | https://fiji.sc/ | | |
| Software, algorithm | RStudio | https://rstudio.com | RRID: SCR_000432 | |
| Software, algorithm | Custom code | This paper | | GitHub |

Table S2. **The average gene expression of highly variable genes in each cluster.** The gene expression levels were normalized by the number of transcripts in each cell as TP10K – transcripts per 10 thousand transcripts. Mean expression of the highly variable genes was then calculated separately for each cluster.

## REFERENCES AND NOTES


1. L. K. Scheffer, C. S. Xu, M. Januszewski, Z. Lu, S. Y. Takemura, K. J. Hayworth, G. B. Huang, K. Shinomiya, J. Maitlin-Shepard, S. Berg, J. Clements, P. M. Hubbard, W. T. Katz, L. Umayam, T. Zhao, D. Ackerman, T. Blakely, J. Bogovic, T. Dolafi, D. Kainmueller, T. Kawase, K. A. Khairy, L. Leavitt, P. H. Li, L. Lindsey, N. Neubarth, D. J. Olbris, H. Otsuna, E. T. Trautman, M. Ito, A. S. Bates, J. Goldammer, T. Wolff, R. Svirskas, P. Schlegel, E. Neace, C. J. Knecht, C. X. Alvarado, D. A. Bailey, S. Ballinger, J. A. Borycz, B. S. Canino, N. Cheatham, M. Cook, M. Dreher, O. Duclos, B. Eubanks, K. Fairbanks, S. Finley, N. Forknall, A. Francis, G. P. Hopkins, E. M. Joyce, S. Kim, N. A. Kirk, J. Kovalyak, S. A. Lauchie, A. Lohff, C. Maldonado, E. A. Manley, S. McLin, C. Mooney, M. Ndama, O. Ogundeyi, N. Okeoma, C. Ordish, N. Padilla, C. M. Patrick, T. Paterson, E. E. Phillips, E. M. Phillips, N. Rampally, C. Ribeiro, M. K. Robertson, J. T. Rymer, S. M. Ryan, M. Sammons, A. K. Scott, A. L. Scott, A. Shinomiya, C. Smith, K. Smith, N. L. Smith, M. A. Sobeski, A. Suleiman, J. Swift, S. Takemura, I. Talebi, D. Tarnogorska, E. Tenshaw, T. Tokhi, J. J. Walsh, T. Yang, J. A. Horne, F. Li, R. Parekh, P. K. Rivlin, V. Jayaraman, M. Costa, G. S. Jefferis, K. Ito, S. Saalfeld, R. George, I. A. Meinertzhagen, G. M. Rubin, H. F. Hess, V. Jain, S. M. Plaza, A connectome and analysis of the adult *Drosophila* central brain. *eLife* **9**, e57443 (2020).

2. V. Svensson, R. Vento-Tormo, S. A. Teichmann, Exponential scaling of single-cell RNA-seq in the past decade. *Nat. Protoc.* **13**, 599–604 (2018).

3. C. Brunet Avalos, G. L. Maier, R. Bruggmann, S. G. Sprecher, Single cell transcriptome atlas of the *Drosophila* larval brain. *eLife* **8**, e50354 (2019).

4. V. Croset, C. D. Treiber, S. Waddell, Cellular diversity in the *Drosophila* midbrain revealed by single-cell transcriptomics. *eLife* **7**, e34550 (2018).

5. K. Davie, J. Janssens, D. Koldere, M. De Waegeneer, U. Pech, L. Kreft, S. Aibar, S. Makhzami, V. Christiaens, C. Bravo Gonzalez-Blas, S. Poovathingal, G. Hulselmans, K. I. Spanier, T. Moerman, B. Vanspauwen, S. Geurs, T. Voet, J. Lammertyn, B. Thienpont, S. Liu, N. Konstantinides, M. Fiers, P. Verstreken, S. Aerts, A single-cell transcriptome atlas of the aging *Drosophila* brain. *Cell* **174**, 982–998.e20 (2018).

6. N. Konstantinides, K. Kapuralin, C. Fadil, L. Barboza, R. Satija, C. Desplan, Phenotypic convergence: Distinct transcription factors regulate common terminal features. *Cell* **174**, 622–635.e13 (2018).

7. Y. Z. Kurmangaliyev, J. Yoo, S. A. LoCascio, S. L. Zipursky, Modular transcriptional programs separately define axon and dendrite connectivity. *eLife* **8**, e50822 (2019).

8. H. Li, J. Janssens, M. De Waegeneer, S. S. Kolluru, K. Davie, V. Gardeux, W. Saelens, F. P. A. David, M. Brbic, K. Spanier, J. Leskovec, C. N. McLaughlin, Q. Xie, R. C. Jones, K. Brueckner, J. Shim, S. G. Tattikota, F. Schnorrer, K. Rust, T. G. Nystul, Z. Carvalho-Santos, C. Ribeiro, S. Pal, S. Mahadevaraju, T. M. Przytycka, A. M. Allen, S. F. Goodwin, C. W. Berry, M. T. Fuller, H. White-Cooper, E. L. Matunis, S. DiNardo, A. Galenza, L. E. O'Brien, J. A. T. Dow, F. C. A. Consortium, H. Jasper, B. Oliver, N. Perrimon, B. Deplancke, S. R. Quake, L. Luo, S. Aerts, D. Agarwal, Y. Ahmed-Braimah, M. Arbeitman, M. M. Ariss, J. Augsburger, K. Ayush, C. C. Baker, T. Banisch, K. Birker, R. Bodmer, B. Bolival, S. E. Brantley, J. A. Brill, N. C. Brown, N. A. Buehner, X. T. Cai, R. Cardoso-Figueiredo, F. Casares, A. Chang, T. R. Clandinin, S. Crasta, C. Desplan, A. M. Detweiler, D. B. Dhakan, E. Dona, S. Engert, S. Floc'hlay, N. George, A. J. Gonzalez-Segarra, A. K. Groves, S. Gumbin, Y. Guo, D. E. Harris, Y. Heifetz, S. L. Holtz, F. Horns, B. Hudry, R. J. Hung, Y. N. Jan, J. S. Jaszczak, G. Jefferis, J. Karkanias, T. L. Karr, N. S. Katheder, J. Kezos, A. A. Kim, S. K. Kim, L. Kockel, N. Konstantinides, T. B. Kornberg, H. M. Krause, A. T. Labott, M. Laturney, R. Lehmann, S. Leinwand, J. Li, J. S. S. Li, K. Li, K. Li, L. Li, T. Li, M. Litovchenko, H. H. Liu, Y. Liu, T. C. Lu, J. Manning, A. Mase, M. Matera-Vatnick, N. R. Matias, C. E. McDonough-Goldstein, A. McGeever, A. D. McLachlan, P. Moreno-Roman, N. Neff, M. Neville, S. Ngo, T. Nielsen, C. E. O'Brien, D. Osumi-Sutherland, M. N. Ozel, I. Papatheodorou, M. Petkovic, C. Pilgrim, A. O. Pisco, C. Reisenman, E. N. Sanders, G. Dos Santos, K. Scott, A. Sherlekar, P. Shiu, D. Sims, R. V. Sit, M. Slaidina, H. E. Smith, G. Sterne, Y. H. Su, D. Sutton, M. Tamayo, M. Tan, I. Tastekin, C. Treiber, D. Vacek, G. Vogler, S. Waddell, W. Wang, R. I. Wilson, M. F. Wolfner, Y. E. Wong, A. Xie, J. Xu, S. Yamamoto, J. Yan, Z. Yao, K. Yoda, R. Zhu, R. P. Zinzen, Fly Cell Atlas: A single-nucleus transcriptomic atlas of the adult fruit fly. *Science* **375**, eabk2432 (2022).

9. M. N. Ozel, F. Simon, S. Jafari, I. Holguera, Y. C. Chen, N. Benhra, R. N. El-Danaf, K. Kapuralin, J. A. Malin, N. Konstantinides, C. Desplan, Neuronal diversity and convergence in a visual system developmental atlas. *Nature* **589**, 88–95 (2021).

10. Y. Z. Kurmangaliyev, J. Yoo, J. Valdes-Aleman, P. Sanfilippo, S. L. Zipursky, Transcriptional programs of circuit assembly in the *Drosophila* visual system. *Neuron* **108**, 1045–1057.e6 (2020).

11. H. Li, F. Horns, B. Wu, Q. Xie, J. Li, T. Li, D. J. Luginbuhl, S. R. Quake, L. Luo, Classifying *Drosophila* olfactory projection neuron subtypes by single-cell rna sequencing. *Cell* **171**, 1206–1220.e22 (2017).

12. H. Li, T. Li, F. Horns, J. Li, Q. Xie, C. Xu, B. Wu, J. M. Kebschull, C. N. McLaughlin, S. S. Kolluru, R. C. Jones, D. Vacek, A. Xie, D. J. Luginbuhl, S. R. Quake, L. Luo, Single-cell transcriptomes reveal diverse regulatory strategies for olfactory receptor expression and axon targeting. *Curr. Biol.* **30**, 1189–1198.e5 (2020).

13. Q. Xie, M. Brbic, F. Horns, S. S. Kolluru, R. C. Jones, J. Li, A. R. Reddy, A. Xie, S. Kohani, Z. Li, C. N. McLaughlin, T. Li, C. Xu, D. Vacek, D. J. Luginbuhl, J. Leskovec, S. R. Quake, L. Luo, H. Li, Temporal evolution of single-cell transcriptomes of *Drosophila* olfactory projection neurons. *eLife* **10**, e63450 (2021).

14. C. N. McLaughlin, M. Brbic, Q. Xie, T. Li, F. Horns, S. S. Kolluru, J. M. Kebschull, D. Vacek, A. Xie, J. Li, R. C. Jones, J. Leskovec, S. R. Quake, L. Luo, H. Li, Single-cell transcriptomes of developing and adult olfactory receptor neurons in *Drosophila*. *eLife* **10**, e63856 (2021).

15. R. Allada, B. Y. Chung, Circadian organization of behavior and physiology in *Drosophila*. *Annu. Rev. Physiol.* **72**, 605–624 (2010).

16. D. Top, M. W. Young, Coordination between differentially regulated circadian clocks generates rhythmic behavior. *Cold Spring Harb. Perspect. Biol.* **10**, e033589 (2018).

17. O. T. Shafer, C. Helfrich-Förster, S. C. Renn, P. H. Taghert, Reevaluation of *Drosophila melanogaster*'s neuronal circadian pacemakers reveals new neuronal classes. *J. Comp. Neurol.* **498**, 180–193 (2006).

18. C. Helfrich-Forster, The circadian clock in the brain: A structural and functional comparison between mammals and insects. *J. Comp. Physiol. A Neuroethol. Sens. Neural Behav. Physiol.* **190**, 601–613 (2004).

19. D. Ma, D. Przybylski, K. C. Abruzzi, M. Schlichting, Q. Li, X. Long, M. Rosbash, A transcriptomic taxonomy of *Drosophila* circadian neurons around the clock. *eLife* **10**, e63056 (2021).

20. Z. Mao, R. L. Davis, Eight different types of dopaminergic neurons innervate the *Drosophila* mushroom body neuropil: Anatomical and physiological heterogeneity. *Front Neural Circuits* **3**, 5 (2009).

21. R. G. Pendleton, A. Rasheed, T. Sardina, T. Tully, R. Hillman, Effects of tyrosine hydroxylase mutants on locomotor activity in *Drosophila*: A study in functional genomics. *Behav. Genet.* **32**, 89–94 (2002).

22. M. Schwaerzel, M. Monastirioti, H. Scholz, F. Friggi-Grelin, S. Birman, M. Heisenberg, Dopamine and octopamine differentiate between aversive and appetitive olfactory memories in *Drosophila*. *J. Neurosci.* **23**, 10495–10502 (2003).

23. K. Kume, S. Kume, S. K. Park, J. Hirsh, F. R. Jackson, Dopamine is a regulator of arousal in the fruit fly. *J. Neurosci.* **25**, 7377–7384 (2005).

24. R. Andretic, B. van Swinderen, R. J. Greenspan, Dopaminergic modulation of arousal in *Drosophila*. *Curr. Biol.* **15**, 1165–1175 (2005).

25. I. Ganguly-Fitzgerald, J. Donlea, P. J. Shaw, Waking experience affects sleep need in *Drosophila*. *Science* **313**, 1775–1781 (2006).

26. O. V. Alekseyenko, Y. B. Chan, R. Li, E. A. Kravitz, Single dopaminergic neurons that modulate aggression in *Drosophila*. *Proc. Natl. Acad. Sci. U.S.A.* **110**, 6151–6156 (2013).

27. T. Ueno, J. Tomita, H. Tanimoto, K. Endo, K. Ito, S. Kume, K. Kume, Identification of a dopamine pathway that regulates sleep and arousal in *Drosophila*. *Nat. Neurosci.* **15**, 1516–1523 (2012).

28. Q. Liu, S. Liu, L. Kodama, M. R. Driscoll, M. N. Wu, Two dopaminergic neurons signal to the dorsal fan-shaped body to promote wakefulness in *Drosophila*. *Curr. Biol.* **22**, 2114–2123 (2012).

29. Y. Aso, D. Sitaraman, T. Ichinose, K. R. Kaun, K. Vogt, G. Belliart-Guerin, P. Y. Placais, A. A. Robie, N. Yamagata, C. Schnaitmann, W. J. Rowell, R. M. Johnston, T. T. Ngo, N. Chen, W. Korff, M. N.

Nitabach, U. Heberlein, T. Preat, K. M. Branson, H. Tanimoto, G. M. Rubin, Mushroom body output neurons encode valence and guide memory-based action selection in *Drosophila*. *eLife* **3**, e04580 (2014).

30. J. O. Gummadova, G. A. Coutts, N. R. Glossop, Analysis of the *Drosophila* Clock promoter reveals heterogeneity in expression between subgroups of central oscillator cells and identifies a novel enhancer region. *J. Biol. Rhythms* **24**, 353–367 (2009).

31. K. C. Abruzzi, A. Zadina, W. Luo, E. Wiyanto, R. Rahman, F. Guo, O. Shafer, M. Rosbash, RNA-seq analysis of *Drosophila* clock and non-clock neurons reveals neuron-specific cycling and novel candidate neuropeptides. *PLOS Genet.* **13**, e1006613 (2017).

32. T. Hashimshony, N. Senderovich, G. Avital, A. Klochendler, Y. de Leeuw, L. Anavy, D. Gennert, S. Li, K. J. Livak, O. Rozenblatt-Rosen, Y. Dor, A. Regev, I. Yanai, CEL-Seq2: Sensitive highly-multiplexed single-cell RNA-Seq. *Genome Biol.* **17**, 77 (2016).

33. S. Parekh, C. Ziegenhain, B. Vieth, W. Enard, I. Hellmann, zUMIs–A fast and flexible pipeline to process RNA sequencing data with UMIs. *Gigascience* **7**, giy059 (2018).

34. C. Helfrich-Forster, The period clock gene is expressed in central nervous system neurons which also produce a neuropeptide that reveals the projections of circadian pacemaker cells within the brain of *Drosophila melanogaster*. *PNAS* **92**, 612–616 (1995).

35. Y. Fujiwara, C. Hermann-Luibl, M. Katsura, M. Sekiguchi, T. Ida, C. Helfrich-Forster, T. Yoshii, The CCHamide1 neuropeptide expressed in the anterior dorsal neuron 1 conveys a circadian signal to the ventral lateral neurons in *Drosophila melanogaster*. *Front. Physiol.* **9**, 1276 (2018).

36. Y. Hamasaka, D. Rieger, M. L. Parmentier, Y. Grau, C. Helfrich-Forster, D. R. Nassel, Glutamate and its metabotropic receptor inDrosophilaclock neuron circuits. *J. Comp. Neurol.* **505**, 32–45 (2007).

37. J. I. Aguilar, M. Dunn, S. Mingote, C. S. Karam, Z. J. Farino, M. S. Sonders, S. J. Choi, A. Grygoruk, Y. Zhang, C. Cela, B. J. Choi, J. Flores, R. J. Freyberg, B. D. McCabe, E. V. Mosharov, D. E. Krantz, J. A. Javitch, D. Sulzer, D. Sames, S. Rayport, Z. Freyberg, Neuronal depolarization drives increased dopamine synaptic vesicle loading via VGLUT. *Neuron* **95**, 1074–1088.e7 (2017).

38. N. Chuhma, H. Zhang, J. Masson, X. Zhuang, D. Sulzer, R. Hen, S. Rayport, Dopamine neurons mediate a fast excitatory signal via their glutamatergic synapses. *J. Neurosci.* **24**, 972–981 (2004).

39. T. S. Hnasko, N. Chuhma, H. Zhang, G. Y. Goh, D. Sulzer, R. D. Palmiter, S. Rayport, R. H. Edwards, Vesicular glutamate transport promotes dopamine storage and glutamate corelease in vivo. *Neuron* **65**, 643–656 (2010).

40. T. S. Hnasko, R. H. Edwards, Neurotransmitter corelease: Mechanism and physiological role. *Annu. Rev. Physiol.* **74**, 225–243 (2012).

41. Y. Aso, R. P. Ray, X. Long, D. Bushey, K. Cichewicz, T. T. Ngo, B. Sharp, C. Christoforou, A. Hu, A. L. Lemire, P. Tillberg, J. Hirsh, A. Litwin-Kumar, G. M. Rubin, Nitric oxide acts as a cotransmitter in a subset of dopaminergic neurons to diversify memory dynamics. *eLife* **8**, e64094 (2019).

42. P. Bou Dib, B. Gnagi, F. Daly, V. Sabado, D. Tas, D. A. Glauser, P. Meister, E. Nagoshi, A conserved role for p48 homologs in protecting dopaminergic neurons from oxidative stress. *PLOS Genet.* **10**, e1004718 (2014).

43. M. Kunst, M. E. Hughes, D. Raccuglia, M. Felix, M. Li, G. Barnett, J. Duah, M. N. Nitabach, Calcitonin gene-related peptide neurons mediate sleep-specific circadian output in *Drosophila*. *Curr. Biol.* **24**, 2652–2664 (2014).

44. J. D. Watson, S. T. Crews, Formation and specification of a *Drosophila* dopaminergic precursor cell. *Development* **139**, 3316–3325 (2012).

45. T. Brody, A. Cravchik, *Drosophila melanogaster* G protein-coupled receptors. *J. Cell Biol.* **150**, F83–88 (2000).

46. M. Schlichting, S. Richhariya, N. Herndon, D. Ma, J. Xin, W. Lenh, K. Abruzzi, M. Rosbash, Dopamine and GPCR-mediated modulation of DN1 clock neurons gates the circadian timing of sleep. *Proc. Natl. Acad. Sci. U.S.A.* **119**, e2206066119 (2022).

47. K. Zinn, E. Ozkan, Neural immunoglobulin superfamily interaction networks. *Curr. Opin. Neurobiol.* **45**, 99–105 (2017).

48. S. E. Mohr, S. G. Tattikota, J. Xu, J. Zirin, Y. Hu, N. Perrimon, Methods and tools for spatial mapping of single-cell RNAseq clusters in *Drosophila*. *Genetics* **217**, iyab019 (2021).

49. H. Li, Single-cell RNA sequencing in *Drosophila*: Technologies and applications. *Wiley Interdiscip. Rev. Dev. Biol.* **10**, e396 (2021).

50. N. Otto, M. W. Pleijzier, I. C. Morgan, A. J. Edmondson-Stait, K. J. Heinz, I. Stark, G. Dempsey, M. Ito, I. Kapoor, J. Hsu, P. M. Schlegel, A. S. Bates, L. Feng, M. Costa, K. Ito, D. D. Bock, G. M. Rubin, G. Jefferis, S. Waddell, Input connectivity reveals additional heterogeneity of dopaminergic reinforcement in *Drosophila*. *Curr. Biol.* **30**, 3200–3211.e8 (2020).

51. O. T. Shafer, G. J. Gutierrez, K. Li, A. Mildenhall, D. Spira, J. Marty, A. A. Lazar, M. P. Fernandez, Connectomic analysis of the *Drosophila* lateral neuron clock cells reveals the synaptic basis of functional pacemaker classes. *eLife* **11**, e79139 (2022).

52. X. Liang, M. C. W. Ho, Y. Zhang, Y. Li, M. N. Wu, T. E. Holy, P. H. Taghert, Morning and evening circadian pacemakers independently drive premotor centers via a specific dopamine relay. *Neuron* **102**, 843–857.e4 (2019).

53. X. Liang, T. E. Holy, P. H. Taghert, Polyphasic circadian neural circuits drive differential activities in multiple downstream rhythmic centers. *Curr. Biol.* **33**, 351–363.e3 (2023).

54. X. Chen, R. Rahman, F. Guo, M. Rosbash, Genome-wide identification of neuronal activity-regulated genes in *Drosophila*. *eLife* **5**, e19942 (2016).

55. P. Xu, S. Berto, A. Kulkarni, B. Jeong, C. Joseph, K. H. Cox, M. E. Greenberg, T. K. Kim, G. Konopka, J. S. Takahashi, NPAS4 regulates the transcriptional response of the suprachiasmatic nucleus to light and circadian behavior. *Neuron* **109**, 3268–3282.e6 (2021).

56. W. J. Dreyer, The area code hypothesis revisited: Olfactory receptors and other related transmembrane receptors may function as the last digits in a cell surface code for assembling embryos. *Proc. Natl. Acad. Sci. U.S.A.* **95**, 9072–9077 (1998).

57. S. J. Smith, U. Sumbul, L. T. Graybuck, F. Collman, S. Seshamani, R. Gala, O. Gliko, L. Elabbady, J. A. Miller, T. E. Bakken, J. Rossier, Z. Yao, E. Lein, H. Zeng, B. Tasic, M. Hawrylycz, Single-cell transcriptomic evidence for dense intracortical neuropeptide networks. *eLife* **8**, e47889 (2019).

58. J. R. Sanes, S. L. Zipursky, Synaptic specificity, recognition molecules, and assembly of neural circuits. *Cell* **181**, 1434–1435 (2020).

59. K. M. Parisky, J. Agosto, S. R. Pulver, Y. Shang, E. Kuklin, J. J. Hodge, K. Kang, X. Liu, P. A. Garrity, M. Rosbash, L. C. Griffith, PDF cells are a GABA-responsive wake-promoting component of the *Drosophila* sleep circuit. *Neuron* **60**, 672–682 (2008).

60. Y. Shang, N. C. Donelson, C. G. Vecsey, F. Guo, M. Rosbash, L. C. Griffith, Short neuropeptide F is a sleep-promoting inhibitory modulator. *Neuron* **80**, 171–183 (2013).

61. M. K. Klose, M. P. Bruchez, D. L. Deitcher, E. S. Levitan, Temporally and spatially partitioned neuropeptide release from individual clock neurons. *Proc. Natl. Acad. Sci. U.S.A.* **118**, e2101818118 (2021).

62. A. Butler, P. Hoffman, P. Smibert, E. Papalexi, R. Satija, Integrating single-cell transcriptomic data across different conditions, technologies, and species. *Nat. Biotechnol.* **36**, 411–420 (2018).

63. A. M. Allen, M. C. Neville, S. Birtles, V. Croset, C. D. Treiber, S. Waddell, S. F. Goodwin, A single-cell transcriptomic atlas of the adult *Drosophila* ventral nerve cord. *eLife* **9**, e54074 (2020).

64. S. C. Renn, J. H. Park, M. Rosbash, J. C. Hall, P. H. Taghert, A pdf neuropeptide gene mutation and ablation of PDF neurons each cause severe abnormalities of behavioral circadian rhythms in *Drosophila*. *Cell* **99**, 791–802 (1999).

65. F. Guo, M. Holla, M. M. Diaz, M. Rosbash, A circadian output circuit controls sleep-wake arousal in *Drosophila*. *Neuron* **100**, 624–635.e4 (2018).

66. B. Deng, Q. Li, X. Liu, Y. Cao, B. Li, Y. Qian, R. Xu, R. Mao, E. Zhou, W. Zhang, J. Huang, Y. Rao, Chemoconnectomics: Mapping chemical transmission in *Drosophila*. *Neuron* **101**, 876–893.e4 (2019).
